# Willingness to receive an annual COVID-19 booster vaccine in the German-speaking D-A-CH region in Europe: A cross-sectional study

**DOI:** 10.1016/j.lanepe.2022.100414

**Published:** 2022-05-29

**Authors:** Jakob Weitzer, Brenda M. Birmann, Ilja Steffelbauer, Martin Bertau, Lukas Zenk, Guido Caniglia, Manfred D. Laubichler, Gerald Steiner, Eva S. Schernhammer

**Affiliations:** a Department of Epidemiology, Center for Public Health, Medical University of Vienna, Kinderspitalgasse 15, Vienna 1090, Austria; b Department of Health Promotion and Prevention, Federal Ministry of the Republic of Austria for Social Affairs, Health, Care and Consumer Protection, Radetzkystraße 2, Vienna 1030, Austria; c Channing Division of Network Medicine, Department of Medicine, Brigham and Women's Hospital and Harvard Medical School, 181 Longwood Avenue, Boston, MA 02115, USA; d Department of Knowledge and Communication Management, Faculty of Business and Globalization, University for Continuing Education Krems, Dr.-Karl-Dorrek-Straße 30, Krems an der Donau 3500, Austria; e Institute of Chemical Technology, Freiberg University of Mining and Technology, Germany; f Konrad Lorenz Institute for Evolution and Cognition Research, Klosterneuburg 3400, Austria; g Complexity Science Hub Vienna, Josefstädter Straße 39, Vienna 1080, Austria; h Arizona State University, Tempe, AZ 85287, USA; i Santa Fe Institute, Santa Fe, NM 87501, USA

**Keywords:** Vaccine hesitancy, Booster dose, Booster jab, COVID19 pandemic

## Abstract

**Background:**

Emergence of new coronavirus variants and waning immunity may necessitate regular COVID-19 vaccine boosters, but empirical data on population willingness for regular vaccination are limited.

**Methods:**

In August 2021, we surveyed 3,067 quota-sampled German-speaking adults residing in the D-A-CH region (Germany, Austria, Switzerland). Using multivariable adjusted ordered logistic regression models we calculated odds ratios (OR) and 95% confidence intervals (95% CIs) to assess factors associated with willingness to vaccinate annually against COVID-19.

**Findings:**

Among 2,480 participants vaccinated or planning to get vaccinated, 82·4% indicated willingness to receive annual COVID-19 boosters. This willingness was higher in Austria (OR=1·47, 95% CI, 1·19–1·82; *p* < 0·001) and Germany (OR=1·98, 95% CI, 1·60–2·45; *p* < 0·001) versus Switzerland and increased with age. Having voted in the last national election (OR_opposition party voters_=1·51, 95% CI=1·18–1·92; *p* = 0·001 and OR_governing party voters_=1·57, 95% CI=1·28–1·93; *p* < 0·001, versus non-voters) and not regularly participating in religious meetings (OR=1·37, 95% CI=1·08–1·73; *p* = 0·009, versus participation at least monthly) were significantly associated with willingness to vaccinate, as was partial (OR=1·97, 95% CI=1·43–2·72; *p* < 0·001) or total (OR=5·20, 95% CI=3·76–7·19; *p* < 0·001) approval of COVID-19 mitigation measures (versus non-approval). By country, Austrians showed the strongest association of voting behavior and mitigation measure approval with willingness to vaccinate.

**Interpretation:**

Targeted promotion programs informed by political and religious engagement and mitigation measure approval are needed to increase willingness to receive regular COVID-19 boosters.

**Funding:**

Medical University of Vienna, Department of Epidemiology, Danube University Krems, Department for Knowledge and Communication Management; Austrian Society of Epidemiology.

**Research in context**

**Evidence before the study**

Even if COVID-19 vaccination or infection rates continue to rise, declining immunity and emergence of new variants will likely require regular boosters, perhaps annually. Despite the known beneficial outcomes of a third (booster) dose of the BNT162b2 mRNA vaccine (Comirnaty; Pfizer-BioNTech) on the risk of infection and severe illness, they are only effective if people are willing to get a booster. While extensive literature documents factors that influence influenza vaccine hesitancy, regarding COVID-19 vaccine hesitancy, less is known. We conducted a literature search in PubMed and Web of Science for papers published before March 2022, using the terms ‘(“vaccine hesitancy” OR “hesitancy”) AND (“COVID-19” OR “SARS-CoV-2” OR “COVID-19 booster”)’. The search terms were restricted to title and abstract. There were no language restrictions. To our knowledge, readiness for annual boosters against COVID-19 in the D-A-CH region has not yet been described, and to date, only a handful of studies worldwide have addressed this important question, though most only assessed willingness to receive a single – not an annual – booster against COVID-19.

**Added value of this study**

This is the first study to assess readiness for annual boosters against COVID-19 in the D-A-CH region (Germany, Austria, Switzerland). Using data of 3,067 quota-sampled German-speaking adults residing in the D-A-CH region, we found that 82·4% indicated willingness to receive annual COVID-19 boosters. This willingness differed somewhat by country (Germany, 77.9%; Austria, 87.1%; Switzerland, 81.6%) and increased with age. Having voted in the last national election and not regularly participating in religious meetings were significantly associated with willingness to vaccinate, as was approval of COVID-19 mitigation measures. Austrians showed the strongest association of voting behavior and mitigation measure approval with willingness to vaccinate.

**Implications of all the available evidence**

Our findings suggest that, in addition to age and political preferences/involvement, religious beliefs or engagement, e.g., frequency of interaction with a religious community, seem to influence willingness to booster annually. The data indicate an overall need for the promotion of vaccine booster acceptance in the D-A-CH region, with promotion efforts tailored especially to young and religious individuals, as well as to those with low approval of COVID-19 mitigation measures and those who did not vote in the last elections. Policy makers, practitioners, and religious communties could use our findings as a starting point for targeted promotion, which might help increase willingness to receive regular COVID-19 boosters.

Alt-text: Unlabelled box

## Introduction

The first cases of coronavirus disease 2019 (COVID-19), caused by the novel coronavirus SARS-CoV-2, were reported in late 2019 in Wuhan, China; On 30 January 2020, the World Health Organization (WHO) declared that the outbreak constituted a Public Health Emergency of International Concern (PHEIC), and by March 11, 2020, it had declared the outbreak a pandemic.^1^ Since then, several SARS-CoV-2 variants have emerged, the most important being, in chronological order: Alpha (B.1.1.7), Beta (B.1.351), Gamma (P.1), Delta (B.1.617.2) and Omicron (B.1.1.529); these variants have led to several waves of infections in Europe including in the D-A-CH region (Germany [Deutschland], Austria, Switzerland [Confoederatio Helvetica]).^2^ By January 2022, the Omicron wave reached the D-A-CH region.^3^, ^4^, ^5^ The dominance of the more infectious Omicron variant and rather low vaccination rates in the region, in which 67%–73% of the total population was fully immunized and 25%–42% had received a booster as of January 1st 2022, favored the emergence of this unparalleled wave of infections.^2^, ^3^, ^4^, ^5^ By February 2022, Austria introduced a vaccine mandate to increase vaccination levels. The mandate was approved by the Austrian Parliament and required vaccination of residents 18 years and older, with exceptions for individuals with pre-existing conditions that would have made the vaccination ineffective or dangerous, for those who had tested positive for COVID-19 within 180 days, and for pregnant women.^6^^,^^7^ Concurrently, similar measures were discussed in Germany.^8^ However, with the decline of the Omicron wave the Austrian vaccine mandate was suspended, and most restrictions were lifted in the D-A-CH region.^6^^,^^9^^,^^10^

Importantly, even if vaccination or infection rates rise sufficiently to achieve some form of “herd immunity,” the possible decline in immunity and emergence of new variants already require, and very likely will continue to require, regular boosters, perhaps annually. Several studies investigating the Delta (B.1.617.2) variant indicated that a third (booster) dose of the BNT162b2 mRNA vaccine (Comirnaty; Pfizer-BioNTech) markedly decreased the risk of infection and severe illness, the latter defined as infection requiring hospital admission or COVID-19-related death.^11^, ^12^, ^13^, ^14^ However, these beneficial outcomes are only effective if people are willing to get a booster.

Extensive systematic research has documented factors that influence influenza vaccine hesitancy.^15^ Low perceived risk of, or susceptibility to, the disease and higher perceived risk of vaccine-related adverse events were associated with lower vaccine uptake. People who perceived the social benefit of the vaccine to be low and those who received low perceived pressure from significant others to vaccinate were less likely to vaccinate. A lack of perceived behavioral control and a negative attitude towards the vaccine were also identified as barriers. Having no history of influenza vaccination and no history of influenza itself were barriers too, as were less frequent interaction with the health system and less frequent receipt of cues to take action. Finally, concerning socioeconomic characteristics, younger age, being female, living alone and being unmarried were identified as barriers for influenza vaccine uptake.^15^ Regarding COVID-19 vaccine hesitancy, less is known.^16^^,^^17^

In June 2020, while several companies worked on the first vaccines against COVID-19, a global survey indicated that 71·5% of the population (68·4% in Germany) would be willing to receive a COVID-19 vaccine.^18^ However, to our knowledge, readiness for annual boosters against COVID-19 in the D-A-CH region has not yet been described, and to date, only a handful of studies worldwide have addressed this important question. Notably, most of those studies only assessed willingness to receive a single – not an annual – booster.^19^, ^20^, ^21^, ^22^, ^23^, ^24^, ^25^, ^26^ Those studies identified several factors that influenced willingness to receive a booster, including trust in the COVID-19 vaccine, in scientists and medical professionals, and in religious leaders, as well as perception of vaccine effectiveness, perceived risk of infection and vaccine side-effects, and less adherence to COVID-19 prevention behaviors.^19^, ^20^, ^21^, ^22^, ^23^, ^24^, ^25^, ^26^ Other factors included mistrust of the pharmaceutical industry, willingness to receive a non-US-manufactured vaccine, being fully immunized, and belief in the effectiveness of mixing/matching different vaccines. The studies also identified sociodemographic and medical history factors associated with willingness to receive a booster, including gender (women were more willing), older age, race, lower education, political conservativeness, obesity, history of chronic disease, and history of SARS-CoV-2 infection.^19^, ^20^, ^21^, ^22^, ^23^, ^24^, ^25^, ^26^ We conducted a survey of adults in the D-A-CH region to investigate these factors in relation to their readiness for annual vaccination against COVID-19.

Earlier studies identified political preference/voting behavior in the last national elections, which took place in Austria in September 2019, in Germany in September 2017 and in Switzerland in October 2019, as an important factor related to vaccine hesitancy.^27^^,^^28^ Another previous study reported that acceptance of an annual COVID-19 vaccine decreased with increasing trust in religious leaders.^23^ Therefore we hypothesized that more frequent participation in religious meetings would be associated with lower willingness to receive an annual COVID-19 booster in our sample.

## Methods

### Study population

Data were collected through a non-probability online survey between July 21, 2021, and August 8, 2021, among 3,067 adults residing in the D-A-CH region, quota-sampled to match the respective population distributions for age, gender, and region of residence (Supplementary Figs. 1–3). Participants had to be age 18 years or older, German-speaking and residing in Germany, Austria or Switzerland. The survey was designed by members of the research team, e.g., the authors of this publication and implemented by the market research institute INTERROGARE, Bielefeld, Germany, using online panels, which are databases - providing broad general population coverage - of potential participants who generally get a reward for filling out a questionnaire. Potential participants are recruited by “open enrollment” and “by invitation only” campaigns via email and online marketing channels. People may choose to answer a survey when they visit their online portal or may receive an email invitation to take part in the survey. The questionnaire (see Supplementary materials) comprised 74 questions on lifestyle, health, and COVID-19 related mitigation measures and behavior, taking an average of 25 min to complete. Response rates were not available, neither were characteristics of non-responders. Participant informed consent was implied by completing the online survey. Data were only accessed and analyzed by members of the research team and did not include participant identifying information. The study was exempt from Institutional Review Board approval according to Federal Regulations 45 CFR 46.10(b).

### Variables

The survey assessed numerous sociodemographic variables, including those listed below:

**Table:** 

• Age • Gender • Country of residence • Citizenship • Migration history • Ethnicity • Educational attainment	• Household income • Living area • Work status • Political preference/ involvement • Participation in religious meetings • Social networks (“contact with a close person I can talk to”)

Additionally, we assessed satisfaction with work and work-life balance. For the latter, we utilized the validated “*Trierer Kurzskala zur Messung von Work-Life Balance*” (TKS-WLB),^29^ which comprised five statements ranked from 1 (totally disagree) to 6 (totally agree) and rendered a score from 5 to 30 for which a higher score indicated better work-life balance.^29^ We also assessed several health factors, including body mass index (BMI), frequency of physical activity (for at least 10 min raising heartbeat or respiratory rate), smoking status, and chronic disease history.

The survey also assessed several personality characteristics. These included optimism, for which we used the validated Life-Orientation-Test revised (LOT-R), which invites responses to six items on a five‐point Likert scale (0–4), rendering a score from 0 to 24 on which higher scores indicate higher optimism.^30^ To assess interpersonal trust, we utilized the validated “*Kurzskala für interpersonales Vertrauen*” (KUSIV3). This instrument features three items that are rated on a scale of 1–5, then summed and divided by three, rendering a final score with 13 possible values between one and five.^31^ The additional personality traits of empathy and perspective taking were assessed with the validated “*Fragebogen für Empathie und Perspektivenübernahme.*” Briefly, empathy and perspective taking are assessed separately, each with nine statements rated on a six-point Likert scale (0–5), rendering a score from 0 to 45 for each trait.^32^ Further, we assessed the “Big Five” personality traits, e.g., conscientiousness, extroversion, agreeableness, openness and neuroticism using the validated Big-Five-Inventory-SOEP (BFI-S) in which three statements per trait are rated on a seven-point Likert scale (1–7), rendering a score from 3 to 21 for each trait.^33^ Lastly, we assessed whether participants considered themselves to be a “No, but” or a “Yes, and” type in conversations. In improvisation, such as in improvisational theater, the “Yes, and” type is considered key for an ability to build spontaneously on previous ideas; it is also considered essential for effective teamwork.^34^ We assessed this variable because we considered that a personality type associated with teamwork – e.g. getting vaccinated to protect others – might also be related to vaccine hesitancy.

Additionally, several variables related to the COVID-19 pandemic were assessed, including history of COVID-19 infection (positive test) and course of the disease, approval of the COVID-19 measures implemented by the government, vaccination against COVID-19, and type of vaccine. We also asked the probability that friends/acquaintances were already vaccinated or would get vaccinated; and, among participants who had not been vaccinated yet, we asked the percentage of the adult population they thought would have to be vaccinated without any severe side-effects (death, disability, long-term disease) before they would change their mind and get vaccinated, as well as other conditions that would increase their willingness to be vaccinated. Finally, participants who were fully or partially immunized or were planning to get vaccinated were asked to rate the following statement (no, does not apply at all; no, does rather not apply; yes, does rather apply; yes, totally applies): I am prepared, if necessary, to get vaccinated every year against COVID-19.

### Statistical methods

Descriptive statistics were used to summarize characteristics of the whole study population and by country of residence (Germany, Austria, Switzerland,). For categorical variables, frequencies were reported and Pearson's chi-squared tests used to test for differences between countries. For continuous variables, median and interquartile range (*IQR*) were reported after Shapiro-Wilk tests indicated that the continuous variables were not normally distributed. We used k-sample equality-of-medians tests to compare findings across countries. We investigated different transformation approaches but did not find one that adequately normalized the continuous variables. Thus, we decided to categorize continuous variables into tertiles based on the total study sample.

For the univariable analyses, we used ordered logistic regression models to calculate odds ratios (OR) and 95% confidence intervals (CI) to investigate factors associated with willingness to get an annual COVID-19 vaccine booster among participants (*n* = 2,479) who were already vaccinated or were planning to vaccinate. Too few participants reported a gender other than man or woman (*n* = 2) to be analyzed separately, and thus those participants were excluded from all analyses.

The following factors were investigated in univariable analyses:

**Table:** 

• Age [18–25; 26–35; 36–45; 46–55; 56–65; older than 65] • Gender [woman; man] • Migration history [first generation; second generation; more than second-generation/none] • Ethnicity [White; other than White] • Educational attainment [No university degree; university degree] • Household income [approx. lowest; intermediate; highest tertile in the country of residence (approximated because the distributions did not permit precise tertile cut-points)] • Living area [urban; rural] • Work status [Full-(part-)time employed; Full-(part-)time self-employed; unemployed; retired; student/in training/civil-/military service; household; temporary contract; permanent contract] • Satisfaction with work [No, does (rather) not apply; Yes, does rather apply; Yes, does apply] • Work-life balance [tertiles] • Political preference/ involvement in the last elections [did not vote; opposition party, governing party] • Participation at religious meetings [at least once a month; less than once a month; never, or almost never] • Contact with a close person I can talk to (except children) [less than once a week; at least once a week; daily] • Self-reported type in conversations [“No, but..” type; “Yes, and…” type]	• Optimism [tertiles] • Interpersonal trust [tertiles] • Empathy [tertiles] • Perspective taking [tertiles] • Conscientiousness [tertiles] • Extroversion [tertiles] • Agreeableness [tertiles] • Openness [tertiles] • Neuroticism [tertiles] • A previous or current COVID-19 infection • Approval of the COVID-19 measures implemented by the government [No, they were unnecessary and unjustified; Yes, partially; Yes, mainly or totally] • Probability that friends/acquaintances are already vaccinated or will get vaccinated [almost all or all are already vaccinated; very likely; rather likely; Nether likely nor unlikely; Unlikely; Very unlikely] • BMI [kg/m^2^] • Frequency of physical activity (for at least 10 min raising heartbeat or respiratory rate) [less than once a week; 1-2 days a week; 3-4 days a week; 5-7 days a week] • Smoking status [never; former; current] • Chronic disease history [asthma; COPD; chronical bronchitis; emphysema; heart attack; angina pectoris or coronary heart disease; cancer; hypertension; stroke or diabetes].

Most of those variables were chosen based on previous research identifying them as factors associated with COVID-19 vaccine hesitancy.^19^, ^20^, ^21^, ^22^, ^23^, ^24^, ^25^, ^26^, ^27^, ^28^ A few (personality traits, work-life-balance, satisfaction with work, self-reported type in conversations) were included based on *a priori* hypotheses by the research team, related in part to our previous study of vaccine hesitancy in Austria.^27^^,^^28^

For the multivariable ordered logistic regression analyses, we initially included all variables that were associated (*p* < 0·05) with willingness to get an annual booster in univariable analyses. We then utilized backward selection to eliminate variables with a *p*-value ≥ 0·05 to arrive at our final models. We followed this approach for the whole sample and for each country separately. Brant Tests (*p* > 0·05) indicated that the parallel regression assumption for ordered logistic regression was not violated, and variance inflation factors (VIF) indicated no multicollinearity (VIF<2). We used missing indicators to represent missing data in the models.

To address the possibility that the participants who reported not voting in the last elections comprised a mix of individuals who chose not to vote and those not legally permitted to vote due to non-citizenship, we conducted sensitivity analyses restricted to legal citizens of the respective countries. A two-sided *p*-value less than 0.05 was considered statistically significant. All data analyses were performed using STATA (version 14.1, 2015, StataCorp LP).

### Role of the funding source

No funding source was involved in the manuscript writing or decision to submit it for publication.

## Results

### Participant characteristics

Of 3,067 survey participants (68·1% fully and 7·0% partially immunized against COVID-19), 48·8% were men. Participant age ranged between 18 and 90 years (mean=48, standard deviation=16.5). Some socioeconomic characteristics, e.g., educational attainment, differed, whereas most personality characteristics were comparable across the country of residence (Supplementary Table 1). Importantly, the quota sampling approach achieved the expected distributions of age, sex and region of residence (Supplementary Figs. 1–3).

Approval of the COVID-19 measures implemented by the government was highest among participants residing in Switzerland (53·3%; compared to Germany, 50·8% and Austria, 49·5%; *p* = 0·317). However, the percentage of participants fully immunized against COVID-19 was highest among residents of Austria (71·6%; compared to Germany, 68·9%, and Switzerland, 63·6%; *p* < 0·001).

Among those who were not yet vaccinated (19·1%), modest percentages of participants indicated that their willingness to vaccinate would potentially increase if they observed that high percentages of the population had been vaccinated without experiencing any severe side effects; for example, 15·7% indicated a potential willingness to be vaccinated if they could observe this for >90% of the population. Further, 13·6% reported that their willingness to vaccinate would increase if the vaccine was free of charge, 22% if they could choose the vaccine themselves, and 8·4% if they would get a voucher. Still, 53·1% of those not planning to get vaccinated indicated that they would not get vaccinated under any circumstances (Supplementary Table 1).

### Factors cross-sectionally associated with willingness to vaccinate annually against COVID-19

Of those who were already vaccinated or planning to get vaccinated (*n* = 2,480), 82·4% said that they were (rather) willing to get vaccinated every year. Willingness to vaccinate increased with age and for participants residing in Germany (versus residing in Switzerland: OR=1·98, 95% CI, 1·16–2·45; *p* < 0·001) or Austria (versus residing in Switzerland: OR=1·47, 95% CI, 1·19–1·82; *p* < 0·001) (Table 1). Those studying, in training, or in civil or military service were more likely to report willingness to vaccinate (OR=1·89, 95% CI, 1·27–2·81; *p* = 0·002) as were unemployed participants (OR= 1·49, 95% CI, 1·01–2·21; *p* = 0·045) and those working in the household (OR= 1·76, 95% CI, 1·17–2·65; *p* = 0·007), than those in full- or part-time employment. Participants that indicated total satisfaction with work were more likely to report willingness to vaccinate (OR=1·902, 95% CI, 1·46–2·47; *p* < 0·001) than those completely or somewhat dissatisfied with work. Better work-life balance was also associated with a higher willingness (Top vs. bottom tertile: OR=1·43, 95% CI, 1·15–1·79; *p* = 0·001).

**Table 1:** Factors cross-sectionally associated with willingness to get an annual COVID-19 booster, if necessary, among participants who already got vaccinated or are planning to get vaccinated in the D-A-CH region (*n* = 2,479).

	No, (rather) not willing to get vaccinated every year (*n* = 435)	Yes, rather willing to get vaccinated every year (*n* = 890)	Yes, willing to get vaccinated every year (*n* = 1,154)				
	n (%)	n (%)	n (%)	OR_crude_ (95% CI)	*p*-value	OR_adj_ (95% CI)^1^	*p*-value^1^
**Age (years)**							
**18**–**25**	81 (18·6)	120 (14·5)	67 (5·8)	Ref.		Ref.	
**26**–**35**	86 (19·8)	162 (18·2)	112 (9·7)	1·35 (1·01–1·80)	0·044	1·76 (1·25–2·48)	0·001
**36**–**45**	99 (22·8)	175 (19·7)	148 (12·8)	1·51 (1·14–2·00)	0·004	1·80 (1·28–2·54)	0·001
**46**–**55**	76 (17·5)	155 (17·4)	205 (17·8)	2·40 (1·81–3·19)	<0·001	2·40 (1·68–3·41)	<0·001
**56**–**65**	65 (14·9)	151 (17·0)	302 (26·2)	3·79 (2·86–5·02)	<0·001	3·14 (2·17–4·55)	<0·001
**≥66**	28 (6·4)	127 (14·3)	320 (27·7)	5·91 (4·41–7·93)	<0·001	3·30 (2·10–5·19)	<0·001
**Gender**							
Women	243 (55·9)	450 (50·6)	519 (45·0)	Ref.			
Men	192 (44·1)	440 (49·4)	635 (55·0)	1·36 (1·17–1·58)	<0·001		
**Country***(living in)*							
Switzerland	169 (38·8)	309 (34·7)	285 (24·7)	Ref.		Ref.	
Germany	112 (25·8)	297 (33·4)	471 (40·8)	1·91 (1·59–2·30)	<0·001	1·98 (1·60–2·45)	<0·001
Austria	154 (35·4)	284 (31·9)	398 (34·5)	1·44 (1·20–1·73)	<0·001	1·47 (1·19–1·82)	<0·001
**Migration history**							
First generation	115 (26·4)	280 (31·5)	280 (24·3)	Ref.			
Second generation	61 (14·0)	96 (10·8)	77 (6·7)	0·66 (0·50–0·87)	0·003		
More than second generation/none	259 (59·6)	514 (57·7)	797 (69·0)	1·32 (1·12–1·57)	0·001		
**Ethnicity**							
Other than White	41 (9·4)	88 (9·9)	70 (6·1)	Ref.			
White	394 (90·6)	802 (90·1)	1,084 (93·9)	1·50 (1·15–1·95)	0·003		
**Educational attainment**							
No university degree	352 (80·9)	675 (75·8)	836 (72·4)	Ref.			
University degree	83 (19·1)	215 (24·2)	318 (27·6)	1·35 (1·14–1·61)	0·001		
**Household income** (tertiles defined at the regional level)^2^							
Approx. lowest tertile	155 (35·6)	328 (36·9)	384 (33·3)	Ref.			
Approx. middle tertile	106 (24·4)	234 (26·2)	299 (25·9)	1·10 (0·91–1·33)	0·324		
Approx. highest tertile	174 (40·0)	328 (36·9)	471 (40·8)	1·13 (0·95–1·34)	0·173		
**Living area**							
Urban	244 (56·1)	489 (54·9)	655 (56·8)	Ref.			
Rural	191 (43·9)	401 (45·1)	499 (43·2)	0·96 (0·83–1·11)	0·590		
**Work status**							
Full- (part-) time employed	213 (49·0)	371 (41·7)	380 (32·9)	Ref.		Ref.	
Full- (part-) time self-employed	32 (7·4)	50 (5·6)	67 (5·8)	1·19 (0·85–1·65)	0·308	0·73 (0·51–1·04)	0·089
Unemployed	24 (5·5)	41 (4·6)	52 (4·5)	1·19 (0·83–1·71)	0·345	1·49 (1·01–2·21)	0·045
Retired	50 (11·5)	186 (20·9)	418 (36·2)	2·84 (2·33–3·45)	<0·001	1·25 (0·92–1·70)	0·159
Student/in training/civil-/military-service	34 (7·8)	64 (7·2)	54 (4·7)	0·90 (0·65–1·23)	0·496	1·89 (1·27–2·81)	0·002
Household	15 (3·4)	40 (4·5)	55 (4·8)	1·60 (1·10–2·33)		1·76 (1·17–2·65)	0·007
Temporary contract	4 (0·9)	24 (2·7)	11 (1·0)	0·95 (0·55–1·66)	0·868	1·52 (0·83–2·80)	0·177
Permanent contract	63 (14·5)	114 (12·8)	117 (10·1)	1·02 (0·80–1·31)	0·844	1·12 (0·87–1·45)	0·386
**Satisfaction with work**							
No, does not or does rather not apply	149 (34·2)	234 (26·3)	244 (21·2)	Ref.		Ref.	
Yes, does rather apply	220 (50·6)	493 (55·4)	508 (44·0)	1·21 (1·01–1·45)	0·035	1·16 (0·94–1·42)	0·163
Yes, does totally apply	66 (15·2)	163 (18·3)	402 (34·8)	2·82 (2·27–3·51)	<0·001	1·90 (1·46–2·47)	<0·001
**Work-Life balance**^3^							
Bottom tertile	190 (43·7)	320 (35·9)	282 (24·4)	Ref.		Ref.	
Middle tertile	140 (32·2)	328 (36·9)	316 (27·4)	1·29 (1·07–1·55)	0·006	1·05 (0·86–1·29)	0·609
Top tertile	105 (24·1)	242 (27·2)	556 (48·2)	2·82 (2·34–3·40)	<0·001	1·43 (1·15–1·79)	0·001
**Main job task**							
Physical work with hands	86 (19·7)	157 (17·6)	131 (11·4)	Ref.			
Mental work with figures/symbols	132 (30·3)	258 (29·0)	274 (23·7)	1·26 (1·00–1·59)	0·052		
Contact/Communication with other people	116 (26·6)	196 (22·0)	213 (18·5)	1·18 (0·92–1·51)	0·183		
Not working	102 (23·4)	279 (31·4)	536 (46·4)	2·53 (2·01–3·17)	<0·001		
**Political preference/involvement** (last elections)							
Did not vote	164 (37·7)	252 (28·3)	182 (15·8)	Ref.		Ref.	
Opposition parties	105 (24·1)	206 (23·2)	322 (27·9)	2·21 (1·79–2·74)	<0·001	1·51 (1·18–1·92)	0·001
Governing parties	166 (38·2)	432 (48·5)	650 (56·3)	2·44 (2·03–2·93)	<0·001	1·57 (1·28–1·93)	<0·001
**Participation at religious meetings**							
At least once a month	91 (20·9)	130 (14·6)	136 (11·8)	Ref.		Ref.	
Less than once a month	69 (15·9)	149 (16·7)	161 (14·9)	1·90 (1·43–2·53)	<0·001	1·22 (0·91–1·63)	0·190
Never, or almost never	275 (63·3)	611 (68·7)	857 (74·3)	2·30 (1·80–2·94)	<0·001	1·37 (1·08–1·73)	0·009
**Contact with a close person (except children) I can talk to**							
Less than once a week	71 (16·3)	106 (11·9)	71 (6·1)	Ref.			
At least once a week	85 (19·5)	164 (18·4)	204 (17·7)	1·31 (1·00–1·72)	0·049		
Daily	279 (64·2)	620 (69·7)	879 (76·2)	1·67 (1·35–2·06)	<0·001		
**In conversations I consider myself a:**							
*“No, but…” type*	131 (30·1)	241 (27·1)	289 (25·0)	Ref.			
*“Yes, and…” type*	304 (69·9)	649 (72·9)	865 (75·0)	1·19 (1·00–1·40)	0·045		
**Optimism**^4^							
Bottom tertile	188 (43·2)	363 (40·8)	346 (30·0)	Ref.			
Middle tertile	154 (35·4)	304 (34·2)	384 (33·3)	1·14 (0·95–1·37)	0·168		
Top tertile	93 (21·4)	223 (25·0)	424 (36·7)	1·72 (1·44–2·05)	<0·001		
**Interpersonal trust**^5^							
Bottom tertile	1831 (42·0)	348 (39·1)	402 (34·8)	Ref.			
Middle tertile	136 (31·3)	279 (31·3)	325 (28·2)	0·98 (0·81–1·20)	0·859		
Top tertile	116 (26·7)	263 (29·6)	427 (37·0)	1·62 (1·31–1·99)	<0·001		
**Empathy**^6^							
Bottom tertile	103 (23·7)	224 (25·2)	413 (35·8)	Ref.			
Middle tertile	90 (20·7)	167 (18·7)	195 (16·9)	1·20 (1·00–1·45)	0·055		
Top tertile	242 (55·6)	499 (56·1)	546 (47·3)	1·55 (1·30–1·86)	<0·001		
**Perspective taking**^6^							
Bottom tertile	158 (36·3)	310 (34·8)	339 (29·4)	Ref.			
Middle tertile	120 (27·6)	240 (27·0)	293 (25·4)	1·11 (0·92–1·35)	0·289		
Top tertile	157 (36·1)	340 (38·2)	522 (45·2)	1·42 (1·19–1·69)	<0·001		
**Conscientiousness**^7^							
Bottom tertile	193 (44·4)	367 (41·2)	311 (27·0)	Ref.			
Middle tertile	100 (23·0)	271 (30·5)	348 (30·1)	1·67 (1·39–2·01)	<0·001		
Top tertile	142 (32·6)	252 (28·3)	495 (42·9)	2·03 (1·70–2·43)	<0·001		
**Extroversion**^7^							
Bottom tertile	131 (30·1)	266 (29·9)	342 (29·6)	Ref.			
Middle tertile	163 (37·5)	348 (39·1)	362 (31·4)	0·86 (0·71–1·03)	0·098		
Top tertile	141 (32·4)	276 (31·0)	450 (39·0)	1·22 (1·01–1·47)	0·038		
**Agreeableness**^7^							
Bottom tertile	188 (43·2)	363 (40·8)	346 (30·0)	Ref.			
Middle tertile	154 (35·4)	304 (34·2)	384 (33·3)	1·28 (1·.08–1·53)	0·005		
Top tertile	93 (21·4)	223 (25·0)	424 (36·7)	2·05 (1·70–2·48)	<0·001		
**Openness**^7^							
Bottom tertile	183 (42·1)	348 (39·1)	402 (34·8)	Ref.			
Middle tertile	136 (31·2)	279 (31·3)	325 (28·2)	1·05 (0·99–1·26)	0·603		
Top tertile	116 (26·7)	263 (29·6)	427 (37·0)	1·48 (1·24–1·77)	<0·001		
**Neuroticism**^7^							
Bottom tertile	103 (23·7)	224 (25·2)	413 (35·8)	Ref.			
Middle tertile	90 (20·7)	167 (18·7)	195 (16·9)	0·61 (0·49–0·76)	<0·001		
Top tertile	242 (55·6)	499 (56·1)	546 (47·3)	0·61 (0·51–·072)	<0·001		
**COVID-19 infection (positive test)**	42 (9·7)	68 (7·6)	62 (5·4)	0·63 (0·47–0·84)	0·002		
**Approval of the COVID-19 measures implemented by the government**							
No, they were unnecessary/ unjustified	79 (18·2)	60 (6·7)	44 (3·8)	Ref.		Ref.	
Yes, partially	218 (50·1)	383 (43·0)	246 (21·3)	1·80 (1·32–2·44)	<0·001	1·97 (1·43–2·72)	<0·001
Yes, mainly or totally	138 (31·7)	447 (50·2)	864 (74·9)	6·21 (4·59–8·41)	<0·001	5·20 (3·76–7·19)	<0·001
**Probability that friends/acquaintances are already vaccinated or will get vaccinated**							
Almost all or all are already ··vaccinated	29 (6·7)	62 (7·0)	142 (12·3)	Ref.		Ref.	
Very likely	93 (21·4)	283 (31·8)	591 (51·2)	1·05 (0·79–1·41)	0·725	0·99 (0·73–1·35)	0·957
Rather likely	119 (27·4)	280 (31·4)	252 (21·8)	0·44 (0·33–0·60)	<0·001	0·53 (0·39–0·73)	<0·001
Nether likely nor unlikely	166 (38·1)	235 (26·4)	151 (13·1)	0·25 (0·19–0·34)	<0·001	0·36 (0·26–0·50)	<0·001
Unlikely	21 (4·8)	22 (2·5)	10 (0·9)	0·16 (0·09–0·29)	<0·001	0·27 (0·14–0·49)	<0·001
Very unlikely	7 (1·6)	8 (0·9)	8 (0·7)	0·30 (0·13–0·69)	<0·001	0·60 (0·25–1·45)	0·259
**BMI** [kg/m²]							
Normal weight [BMI≥18·5 & <25]	198 (49·8)	389 (47·5)	417 (38·5)	Ref.			
Underweight [BMI<18·5]	18 (4·5)	31 (3·8)	20 (1·8)	0·63 (9·41–0·99)	0·043		
Overweight [BMI≥25 & <30]	104 (26·1)	269 (32·9)	380 (35·1)	1·45 (1·21–1·73)	<0·001		
Obesity [BMI≥30]	78 (19·6)	129 (15·8)	267 (24·6)	1·67 (1·35–2·07)	<0·001		
**Frequency of physical activity done for at least 10 min which raises the heartbeat or the respiratory rate**							
Less than once a week	76 (17·5)	150 (16·8)	260 (22·6)	Ref.			
1-2 days a week	122 (28·0)	263 (29·6)	262 (22·7)	0·64 (0·51–0·80)	<0·001		
3-4 days a week	131 (30·1)	252 (28·3)	289 (25·0)	0·68 (0·54–0·85)	0·001		
5-7 days a week	106 (24·4)	225 (25·3)	343 (29·7)	0·92 (0·73–1·15)	0·464		
**Smoking status**							
Never	192 (44·2)	378 (42·5)	460 (39·9)	Ref.			
Former	108 (24·8)	240 (27·0)	358 (31·0)	1·27 (1·06–1·53)	0·009		
Current	135 (31·0)	272 (30·5)	336 (29·1)	1·03 (0·86–1·22)	0·780		
**Chronic disease**^8^	140 (32·2)	330 (37·1)	612 (53·0)	1·99 (1·71–2·32)	<0·001	1·28 (1·07–1·53)	0·007

1 mutually adjusted for all variables for which adjusted odds ratios with 95% confidence intervals and adjusted *p*-values are reported.

2 household income tertiles were approximated because the distributions did not permit precise tertile cut-points.

3 TKS-WLB^29^

4 LOT-R^30^

5 KUSIV3^31^

6 questionnaire for empathy and perspective taking, German version^32^.

7 BFI-S^33^.

8 asthma, COPD, chronical bronchitis, emphysema, heart attack, angina pectoris or coronary heart disease, cancer, hypertension, stroke or diabetes.

Willingness to vaccinate annually was higher among those who had voted either for an opposition party (OR=1·51, 95% CI, 1·18–1·92; *p* = 0·001) or a governing party (OR=1·57, 95% CI, 1·28–1·93) compared to those who had not voted in the last elections. Participants who never or almost never participate in religious meetings showed higher willingness (OR=1·37, 95% CI, 1·08–1·73; *p* = 0·009) compared to those participating at least once a month in such meetings. Considering personality characteristics, we found no association with willingness to vaccinate in the multivariable models.

Regarding approval of the COVID-19 measures implemented by the government, those who partially (OR=1·97, 95% CI, 1·43–2·72; *p* < 0·001) or totally (OR=5·20, 95% CI, 3·76–7·190; *p* < 0·001) approved of them were more likely to be willing to vaccinate compared to those who did not approve of them. Those participants who reported that it was rather likely (OR=0·53, 95% CI, 0·39–0·73; *p* < 0·001), neither likely nor unlikely (OR=0·36, 95% CI, 0·26–0·50; *p* < 0·001) or unlikely (OR=0·27, 95% CI, 0·14–0·49; *p* < 0·001) that their friends/acquaintances were already vaccinated or were going to get vaccinated were less likely to report willingness to vaccinate compared to those who reported that most or all of their friends/acquaintances were already vaccinated. Finally, those who reported to have a chronic disease showed higher willingness (OR=1·28, 95% CI, 1·07–1·53; *p* = 0·007) (Table 1).

### Country-specific factors cross-sectionally associated with willingness to vaccinate annually against COVID-19

When stratifying by country, only higher approval of COVID-19 measures implemented by the government and higher satisfaction with work were cross-sectionally associated with higher willingness to vaccinate in all three countries. Reporting that friends/acquaintances were rather likely, nether likely nor unlikely, or unlikely to be already vaccinated or willing to get vaccinated was associated with less willingness to vaccinate annually in all three countries (Supplementary Tables 2–4).

Having voted in the last elections was associated with higher willingness to vaccinate in Austria (voters for opposition parties: OR=1·91, 95% CI, 1·23-2·98; *p* = 0·004; voters for governing parties: OR=2·21, 95% CI, 1·47–3·334; *p* < 0·001; each compared to non-voters; Supplementary Table 3), and less strongly in Switzerland (voters for governing parties: OR= 1·41, 95% CI, 1·03–1·93; *p* = 0·033; Supplementary Table 4), but not in Germany (Supplementary Table 2). In the sensitivity analyses restricted to legal citizens, the associations of having voted with willingness to receive annual boosters became somewhat stronger in Austria (voters for opposition parties: OR=2·04, 95% CI, 1·20–3·46; *p* = 0·008; voters for governing parties: OR=2·43, 95% CI, 1·48–3·99; *p* < 0·001; each compared to non-voters) and in the Swiss sample (voters for opposition parties: OR=1·80, 95% CI, 1·06–3·08; *p* = 0·031; voters for governing parties: OR=1·49, 95% CI, 1·06–2·10; *p* = 0·021; each compared to non-voters).

Age was associated with higher willingness to receive an annual booster in Germany and Austria, but not in Switzerland. In contrast, Switzerland was the only country in which reporting to have chronic disease, higher levels of perspective taking and higher levels of work-life balance were associated with a higher willingness to receive annual booster shots (Supplementary Table 4). In Germany, those who reported to have no or a more than second generation migration history showed higher willingness to vaccinate (compared to those with a first generation migration history), and willingness to vaccinate increased with income. Further, those in Germany who almost never or never participate in religious meetings showed higher willingness than those who participate at least once a month, and those more frequently having contact with a close person they could talk to showed higher willingness. Lastly, German participants with obesity reported higher willingness than those with normal weight. However, those associations were not observed in Switzerland or Austria.

In Austria (Supplementary Table 3), men were more likely than women to be willing to vaccinate annually (OR=1·50, 95% CI, 1·11–2·03; *p* = 0·008). No associations with gender were observed in Switzerland and Germany.

## Discussion

In the present sample of the D-A-CH region, 82·4% of those who were already vaccinated or planned to get vaccinated were willing or rather willing to get vaccinated annually against COVID-19. Although this indicates a high willingness to get an annual COVID-19 booster shot, the fact that 17·6% are not or rather not willing to get a booster and that 19·1% of the sample indicated an overall hesitancy to vaccinate for the first time is concerning and underscores the need for further targeted efforts to increase willingness to vaccinate in the D-A-CH region.

Previous studies among different populations have reported comparable results, although the comparisons are limited by the fact that some of those studies focused on a single rather than an annual booster dose.^19^^,^^23^^,^^25^ In a study of 2,427 Polish adults conducted in September 2021 (before a recommendation for a booster against COVID-19 was issued in Poland), 71% of the fully immunized participants reported willingness to receive a single COVID-19 vaccine booster; the main reasons for refusing a booster included safety concerns, side effects experienced after the previous dose, and not seeing the necessity for a booster.^19^ According to a survey administered in December 2020 and February to March 2021 to 5,256 adults in the US, 58·5% definitely or most likely intended to get a single COVID-19 vaccine booster, and 20·5% reported that they might get the booster.^25^ Among 744 Chilean adults recruited for a survey between May and June 2021, of whom 93·4% had received at least one dose of a COVID-19 vaccine, 88·2% reported that they would accept a hypothetical COVID-19 vaccine booster, but only 57·8% would accept an annual COVID-19 vaccination.^23^ However, a study in Denmark reported that in September 2021, 90% of the vaccinated Danish population were willing to receive additional COVID-19 booster shots if needed.^35^

An additional four studies focused on health care personnel, who are generally expected to show higher vaccine acceptance than the general population. In a survey of 496 Japanese medical students (89·1% fully immunized), the willingness to receive a booster amounted to 84·5%; relaxation of mobility restrictions, trust in vaccines, and concerns about waning immunity were the main drivers of their willingness to vaccinate.^20^ Among 316 US medical students (95·3% vaccinated), 88·9% were amenable to a single booster dose.^21^ Among 1,358 US health care workers, 83·6% reported they would accept a hypothetical yearly booster; however, this percentage shrank to 13·8% among those who reported vaccine hesitancy regarding the first and second dose.^24^ Finally, in a sample of 1,279 Saudi Arabian health care workers only 55·3% reported willingness to receive a single booster dose.^22^ Again, most of the aforementioned studies assessed willingness to receive a single booster, not willingness to receive an annual booster, limiting their comparability to the present study. The limited previous research assessing both indicates that willingness to receive a single booster dose could be much higher than willingness to receive an annual booster.^23^

In the current study, older participants, those residing in Austria or Germany and those highly satisfied with work were more likely to report willingness to vaccinate annually. In the Polish study, willingness to receive a single booster dose was significantly higher in older subjects, obese individuals, in those with chronic diseases, and among women.^19^ In contrast, in our study gender and BMI, when looking at the whole D-A-CH region, were not significantly associated with willingness to vaccinate annually, nor was a report of a previous COVID infection. However, in the Polish sample, those with an infection prior to vaccination were less frequently in favor of the single booster dose, and those infected after having received one dose mostly rejected the idea of a booster.^19^

In our study sample, willingness to vaccinate annually was higher among those who had voted, whether for an opposition or a governing party, compared to those who had not voted in the last elections, which took place in Austria in September 2019, in Germany in September 2017 and in Switzerland in October 2019. One potential explanation might be that vaccine promotion programs and information on the necessity for a COVID-19 vaccination in general did not reach or did not convince those who did not vote in the last elections. In previous surveys we had identified political preference/involvement as an important factor associated cross-sectionally with hesitancy regarding the first COVID-19 vaccine dose in Austria.^27^^,^^28^ Specifically, in an Austrian sample, we found that those having voted for an opposition party (OR=2·06, 95% CI= 1·44–2·95) and those who did not vote (OR=2·25, 95% CI= 1·53–3·30) in the last elections showed higher vaccine hesitancy compared to those who had voted for a governing party, independent of other factors.^28^ The results from our present study are consistent with the earlier findings in the sense that participants who had not voted in the last elections had higher hesitancy for both the first vaccine and annual booster shots, compared to those who had voted for a governing party, although the findings differed somewhat by country in the present study. Of note, the present survey was not designed to be representative of the D-A-CH region regarding citizenship and therefore the right to vote, which may have introduced selection bias (e.g., 17.1% of persons living in Austria do not hold citizenship, but only less than 10% in our sample indicated that they did not hold Austrian citizenship).^36^

Independent of former voting behavior, those who partially or totally approved of the COVID-19 mitigation measures implemented by the government were more likely to be willing to vaccinate annually compared to those who did not approve of them. Apart from political leaders and movements, scientists and medical professionals can play a major role in increasing booster acceptance and uptake. The Chilean survey found that those with higher trust in medical professionals and scientists reported a 2·8-fold (95% CI=1·5–5·0) higher willingness to receive a first booster and a 2·2-fold (95% CI=1·6–3·1) higher willingness to accept an annual booster shot.^23^

In addition to political preferences/involvement and trust in scientists and medical professionals, religious beliefs or engagement seem to influence willingness to booster annually. In our study, when analyzing the German sample separately, participants who never or almost never participate in religious meetings showed higher willingness to regularly vaccinate compared to those participating at least once a month in such meetings. We note that our survey did not specify the type of religious beliefs or affiliations of the participants who attended meetings regularly, and thus we cannot link this observation to any specific religious belief or group. A more detailed study would be necessary to elucidate this finding. Generally consistent with our findings, another study reported that willingness to accept an annual booster decreased by 30% (95% CI=10%–40%) per 1-unit increase in trust in religious leaders.^23^ In addition, in our study, those who reported anything other than a high likelihood that their friends/acquaintances were already vaccinated or going to get vaccinated were less likely to report willingness to vaccinate annually. This suggests that there may be social network clusters in the population in which vaccine hesitancy is more pronounced. Finally, our data concerning willingness to accept the first vaccine dose suggested that around 50% of those not immunized and not planning to get immunized might be willing to vaccinate if, e.g., they could choose the vaccine or would get a voucher. These results might also apply to willingness to receive an annual booster shot; unfortunately, we did not assess these enabling factors in relation to willingness to get an annual booster.

In our analyses, several personality traits also remained associated with willingness to vaccinate in multivariable models, albeit more weakly than the factors previously discussed. For example, extroversion and openness were positively associated with willingness to vaccinate, and neuroticism inversely. These findings are in line with previous literature that reported certain psychological dispositions and personality traits to be associated with willingness to receive a COVID-19 vaccine in general.^16^

Limitations of our study include the cross-sectional design and that we only assessed the willingness to receive an annual booster among participants who were already vaccinated or were planning to get vaccinated. Although the questionnaire included validated instruments, the questionnaire in its entirety was not piloted before full implementation, limiting our capacity to comment on the appropriateness of the questions. Of note, the categories offered for the question assessing work status were not mutually exclusive, but participants were only able to choose one category. Consequently, our characterization of work status may be misclassified. Additionally, our assessment of chronic disease history did not include some prevalent chronic diseases; most notably, we did not ask about dementia because patients with dementia were likely underrepresented in the quota sampling panels due to the potential unreliability of their responses to the survey. We did not collect data on participant history of vaccine side-effects or perception of risk of the disease. These and other unmeasured factors may be sources of residual confounding, and bias or non-differential misclassification of some self-reported variables may have influenced our findings. Further, the use of an online survey might have limited the representativeness of our sample and the generalizability of our results, including the inclusion of a lower number of non-citizens compared to population averages. In addition, the sample was designed to represent the age, gender, and regional distributions in the D-A-CH region but not national distributions of other characteristics, such as citizenship. Strengths of our study include its large sample size and the representativeness of the sample with regard to the age, gender, and regional population distributions within the respective D-A-CH countries. As such, our findings add to the limited evidence base regarding the willingness to receive an annual COVID-19 booster.

In summary, our study suggests an overall need for the promotion of vaccine booster acceptance in the D-A-CH region, with promotion efforts tailored especially to young and religious individuals, as well as to those with low satisfaction with work and work-life balance, those with low approval of COVID-19 mitigation measures and those who did not vote in the last elections.

## Contributors

JW, BMB, IS, MB, LZ, GC, MDL, GS, and ESS designed the study, ES and GS are responsible for data collection, JW cleaned the data and performed the analyses, conducted literature searches and wrote the first draft of the manuscript. JW, BMB, IS, MB, LZ, GC, MDL, GS, and ESS discussed the results and interpretation of the data. JW, BMB, IS, MB, LZ, GC, MDL, GS, and ESS reviewed and approved the manuscript. JW, GS and ES had access to the raw data.

## Declaration of interests

JW received funding from the Austrian Society of Epidemiology. BMB reports that her institution (Brigham and Women's Hospital, Boston, MA, USA) received research grant funding from the US National Institutes of Health and from the American Institute for Cancer Research (AICR) to support research projects that she leads and that are unrelated to this manuscript (cancer etiology studies). She is also has been an unpaid member of the Scientific Advisory Panel of the Oliver Foundation (Key Biscayne, FL) since 2015 to help guide their work on pediatric cancer prevention. IS reports royalties from book publications and Honoraria (about 2-3 times per year) for invited lectures not exceeding on the average € 1000 per year. MB reports to have received funding from the German Federal Ministry of Science and Education, German Federal Ministry of Economic affairs, Saxonian Ministry of Science and the Arts, EU H-2020 and honoraria for reviews from European public funding organizations. MB also reports patents DE102021115850.8; DE102020134133.4; DE102020107138.8; DE102018105449.1; and he is Member of the Executive committee of the Saxonian Academy of Sciences, Secretary General for the technical sciences in the Saxonian Academy of Sciences, Head of DECHEMA expert group “Raw Materials”, as well as Director of Recycling unit at Fraunhofer Institute for Ceramic Technologies and Systems IKTS; Fraunhofer Technology Center for High-Performance Materials THM, Am St.-Niclas-Schacht 13, 09599 Freiberg, Germany. LZ reports small shares in a stock portfolio < 500 EUR. MDL reports funding from the NSF (USA), small amounts of royalties for several published books, and he is Advisory boards (KLI; Complexity Science Hub, Vienna, TU Graz; TISE). ESS reports funding from the National Institute of Health, FFG, FWF, H-2020, and received honoraria (about 2-3 times per year, each never >1000 Euro) for invited lectures. She is member on the Advisory board of trustees of the FWF (Austrian Science Fund), and the Scientific Advisory Board for the Annual Houska Award; honorary board member of the Austrian Cancer Help; scientific advisory board member for Cochrane Austria; advisory board member of the Austrian Scientific Community and member of Board of Trustees “Sparkling Science” Funding, Ministry of Health Austria; as well as ad hoc NIH study section member and Horizon Europe Scientific Review Panel member.

## Appendix Supplementary materials

Image, application 1

Image, application 2
