# Supplementary material for: Willingness to receive an annual COVID-19 booster vaccine in the German-speaking D-A-CH region in Europe: A cross-sectional study
Source: Lancet Reg Health Eur. 2022 May 29;18:100414. doi: 10.1016/j.lanepe.2022.100414 (PMC9148542; doi:10.1016/j.lanepe.2022.100414)
Supplement: Supplementary file 1 [file mmc1.docx]

**Supplementary figures: Comparisons of quota-sampled participants and national populations in the D-A-CH study region**

**Supplementary figure 1. Comparisons of the age distributions of quota-sampled participants vs national populations by country for (A) Germany, (B) Austria, and (C) Switzerland.**

Source for the German age distribution: <https://www-genesis.destatis.de/genesis/online?operation=abruftabelleBearbeiten&levelindex=2&levelid=1650476823843&auswahloperation=abruftabelleAuspraegungAuswaehlen&auswahlverzeichnis=ordnungsstruktur&auswahlziel=werteabruf&code=12111-0002&auswahltext=&werteabruf=Werteabruf#abreadcrumb>

Source for the Austrian age distribution: <http://www.statistik.at/web_de/statistiken/menschen_und_gesellschaft/bevoelkerung/bevoelkerungsstruktur/bevoelkerung_nach_alter_geschlecht/index.html>

Source for the Swiss age distribution: <https://www.pxweb.bfs.admin.ch/pxweb/de/px-x-0102010000_101/px-x-0102010000_101/px-x-0102010000_101.px/table/tableViewLayout2/>

**Supplementary figure 2. Comparisons of the gender distributions of quota-sampled participants vs national populations by country**

Source for the German gender distribution: <https://www.destatis.de/DE/Themen/Gesellschaft-Umwelt/Bevoelkerung/Bevoelkerungsstand/_inhalt.html;jsessionid=41BC52C61A75401AC28A8FEF71B00CFE.live712>

Source for the Austrian gender distribution: <http://www.statistik.at/wcm/idc/idcplg?IdcService=GET_PDF_FILE&RevisionSelectionMethod=LatestReleased&dDocName=023468>

Source for the Swiss gender distribution: <https://www.pxweb.bfs.admin.ch/pxweb/de/px-x-0102010000_101/px-x-0102010000_101/px-x-0102010000_101.px/table/tableViewLayout2/>

**Supplementary figure 3. Comparisons of the regional distributions of quota-sampled participants vs national populations by country for (A) Germany and (B) Austria** [Comparable data were not readily available for Switzerland]

Source for the German regional distribution: <https://de.wikipedia.org/wiki/Liste_der_deutschen_Bundesl%C3%A4nder_nach_Bev%C3%B6lkerung>

Source for the Austrian regional distribution: <http://www.statistik.at/wcm/idc/idcplg?IdcService=GET_PDF_FILE&RevisionSelectionMethod=LatestReleased&dDocName=023470>
