# Supplementary material for: Willingness to receive an annual COVID-19 booster vaccine in the German-speaking D-A-CH region in Europe: A cross-sectional study
Source: Lancet Reg Health Eur. 2022 May 29;18:100414. doi: 10.1016/j.lanepe.2022.100414 (PMC9148542; doi:10.1016/j.lanepe.2022.100414)
Supplement: Supplementary file 2 [file mmc2.docx]

| **Supplementary Table 1** Selected characteristics of the study sample in total and by country of residence in the D-A-CH region of Europe | | | | |  |
| --- | --- | --- | --- | --- | --- |
|  | Total  (n=3,067) | Switzerland  (n=1,023) | Germany  (n=1,025) | Austria  (n=1,019) |  |
|  | *n (%)* | *n (%)* | *n (%)* | *n (%)* | *p-value [1]* |
| **Age, years** |  |  |  |  |  |
| **18-25** | 339 (11·0) | 112 (10·9) | 98 (9·6) | 129 (12·7) |  |
| **26-35** | 477 (15·6) | 170 (16·6) | 150 (14·6) | 157 (15·4) |  |
| **36-45** | 570 (18·6) | 206 (20·1) | 177 (17·3) | 187 (18·4) |  |
| **46-55** | 555 (18·1) | 173 (16·9) | 185 (18·0) | 197 (19·3) |  |
| **56-65** | 596 (19·4) | 191 (18·7) | 207 (20·2) | 198 (19·4) |  |
| **≥66** | 530 (17·3) | 171 (16·7) | 208 (20·3) | 151 (14·8) | 0·034 |
| **Gender** |  |  |  |  |  |
| Women | 1,567 (51·1) | 515 (50·3) | 531 (51·8) | 521 (51·1) |  |
| Men | 1,498 (48·8) | 508 (49·7) | 492 (48·0) | 498 (48·9) |  |
| Other | 2 (0·1) | 0 | 2 (0·2) | 0 | 0·344 |
| **Migration history** |  |  |  |  |  |
| First generation | 833 (27·2) | 264 (25·8) | 345 (33·7) | 224 (22·0) |  |
| Second generation | 297 (9·7) | 138 (13·5) | 67 (6·5) | 92 (9·0) |  |
| More than second generation/none | 1,937 (63·1) | 621 (60·7) | 613 (59·8) | 703 (69·0) | <0·001 |
| **Ethnicity** |  |  |  |  |  |
| White | 2,806 (91·5) | 917 (89·6) | 958 (93·5) | 931 (91·4) |  |
| Other than White | 261 (8·5) | 106 (10·4) | 67 (6·5) | 88 (8·6) | 0·008 |
| **Educational attainment** |  |  |  |  |  |
| University degree | 730 (23·8) | 200 (19·6) | 330 (32·2) | 200 (19·6) |  |
| No university degree | 2,337 (76·2) | 823 (80·4) | 695 (67·8) | 819 (80·4) | <0·001 |
| **Household income** (tertiles defined at the regional level) _[2]_ |  |  |  |  |  |
| Approx. lowest tertile | 1,141 (37·2) | 342 (33·4) | 389 (38·0) | 410 (40·2) |  |
| Approx. middle tertile | 787 (25·7) | 316 (30·9) | 236 (23·0) | 235 (23·1) |  |
| Approx. highest tertile | 1,139 (37·1) | 365 (35·7) | 400 (39·0) | 374 (36·7) | <0·001 |
| **Living area** |  |  |  |  |  |
| Rural | 1,409 (45·9) | 587 (57·4) | 361 (35·2) | 461 (45·2) |  |
| Urban | 1,658 (54·1) | 436 (42·6) | 664 (64·8) | 558 (54·8) | <0·001 |
| **Work status** |  |  |  |  |  |
| Full- (part-) time employed | 1,222 (39·8) | 440 (43·0) | 399 (38·9) | 383 (37·6) |  |
| Full- (part-) time self-employed | 203 (6·6) | 69 (6·7) | 62 (6·1) | 72 (7·1) |  |
| Unemployed | 158 (5·2) | 41 (4·0) | 50 (4·9) | 67 (6·6) |  |
| Retired | 745 (24·3) | 212 (20·7) | 270 (26·3) | 263 (25·8) |  |
| Student/in training/civil-/military-service | 174 (5·7) | 58 (5·7) | 46 (4·5) | 70 (6·9) |  |
| Household | 150 (4·9) | 63 (6·2) | 56 (5·5) | 31 (3·0) |  |
| Temporary contract | 57 (1·9) | 20 (2·0) | 21 (2·1) | 16 (1·6) |  |
| Permanent contract | 358 (11·6) | 120 (11·7) | 121 (11·8) | 117 (11·4) | 0·001 |
| **Satisfaction with work** |  |  |  |  |  |
| No, does not or does rather not apply | 811 (26·4) | 198 (19·4) | 314 (30·6) | 299 (29·4) |  |
| Yes, does rather apply | 1,489 (48·6) | 531 (51·9) | 486 (47·4) | 472 (46·3) |  |
| Yes, does totally apply | 767 (25·0) | 294 (28·7) | 225 (22·0) | 248 (24·3) | <0·001 |
| **Work-Life balance** _[3]_  *median (IQR)* | *21 (17-25)* | *21 (18-25)* | *21 (17-25)* | *21 (17-25)* | 0·435 |
| Bottom tertile | *991 (32*·3*)* | *289 (28*·2*)* | *339 (33*·1*)* | *363 (35*·6*)* |  |
| Middle tertile | *976 (31*·8*)* | *359 (35*·1*)* | *326 (31*·8*)* | *291 (28*·6*)* |  |
| Top tertile | *365 (35*·9*)* | *375 (36*·7*)* | *360 (35*·1*)* | *365 (35*·8*)* | 0·003 |
| **Main job task** |  |  |  |  |  |
| Physical work with hands | 510 (16·6) | 187 (18·3) | 155 (14·1) | 168 (16·5) |  |
| Mental work with figures/symbols | 791 (25·8) | 269 (26·3) | 282 (27·5) | 240 (23·5) |  |
| Contact/Communication with other people | 678 (22·1) | 250 (24·4) | 196 (19·1) | 232 (22·8) |  |
| Not working | 1,088 (35·5) | 317 (31·0) | 392 (38·2) | 379 (37·2) | <0·001 |
|  |  |  |  |  |  |
|  |  |  |  |  |  |
|  |  |  |  |  |  |
|  |  |  |  |  |  |
|  |  |  |  |  |  |
| **Political preference/involvement** (last elections) | |  |  |  |  |
| Did not vote | 876 (28·5) | 485 (47·4) | 196 (19·1) | 195 (19·1) |  |
| Opposition parties | 775 (25·3) | 115 (11·2) | 333 (32·5) | 327 (32·1) |  |
| Governing parties | 1,416 (46·2) | 423 (41·4) | 496 (48·4) | 497 (48·8) | <0·001 |
| **Participation at religious meetings** | |  |  |  |  |
| At least once a month | 415 (13·5) | 132 (12·9) | 158 (15·4) | 125 (12·3) |  |
| Less than once a month | 470 (15·3) | 161 (15·7) | 127 (12·4) | 182 (17·8) |  |
| Never, or almost never | 2,182 (71·2) | 730 (71·4) | 740 (72·2) | 712 (69·9) | 0·005 |
| **Contact with a close person (except children) that I can talk to** | |  |  |  |  |
| Less than once a week | 308 (10·1) | 105 (10·3) | 129 (12·6) | 74 (7·3) |  |
| At least once a week | 565 (18·4) | 210 (20·5) | 191 (18·6) | 164 (16·1) |  |
| Daily | 2,194 (71·5) | 708 (69·2) | 705 (68·8) | 781 (76·6) | <0·001 |
| **In conversations I consider myself a:** | |  |  |  |  |
| *“No, but…” type* | 864 (28·2) | 285 (27·9) | 302 (29·5) | 277 (27·2) |  |
| *“Yes, and…” type* | 2,203 (71·8) | 738 (72·1) | 723 (70·5) | 742 (72·8) | 0·500 |
| **Optimism** _[4]_ *median (IQR)* | *14 (12-17)* | *14 (12-17)* | *13 (11-16)* | *14 (12-17)* | <0·001 |
| Bottom tertile | *1,*150 (37·5) | *335 (32*·7*)* | *453 (44*·2*)* | *362 (35*·5*)* |  |
| Middle tertile | *819 (26*·7*)* | *278 (27*·2*)* | *284 (27*·7*)* | *257 (25*·2*)* |  |
| Top tertile | *1,*098 (35·8) | *410 (*40·1*)* | *288 (28*·1*)* | *400 (39*·3*)* | <0·001 |
| **Interpersonal trust** _[5]_ *median (IQR)* | *3 (2*·67-3·67*)* | *3*·33 (2·67-3·67) | *3 (2*·33-3·67*)* | *3 (2*·*67-3*·*67)* | <0·001 |
| Bottom tertile | *710 (23*·1*)* | *200 (19*·5*)* | *259 (25*·3*)* | *251 (24*·6*)* |  |
| Middle tertile | *1,341 (43*·7*)* | *450 (44*·0*)* | *442 (43*·1*)* | *449 (44*·1*)* |  |
| Top tertile | *1,016 (33*·2*)* | *373 (36*·5*)* | *324 (31*·6*)* | *319 (31*·3*)* | 0·007 |
| **Empathy** _[6]_ *median (IQR)* | *30 (25-35)* | *31 (26-35)* | *30 (24-35)* | *30 (24-36)* | 0·128 |
| Bottom tertile | *964 (31*·4*)* | *277 (27*·1*)* | *351 (34*·2*)* | *336 (33*·0*)* |  |
| Middle tertile | *926 (30*·2*)* | *339 (33*·1*)* | *301 (29*·4*)* | *286 (28*·1) |  |
| Top tertile | *1,177 (38*·4*)* | *407 (39*·8*)* | *373 (36*·4*)* | *397 (38*·9) | 0·003 |
| **Perspective taking** _[6]_*median (IQR)* | *28 (24-34)* | *29 (24-34)* | *28 (23-35)* | *28 (23-33)* | 0·141 |
| Bottom tertile | *1,003 (32*·7*)* | *302 (29*·5*)* | *347 (33*·8*)* | *354 (34*·7*)* |  |
| Middle tertile | *810 (26*·4*)* | *284 (27*·8*)* | *264 (25*·8*)* | *262 (25*·7*)* |  |
| Top tertile | *1,254 (40*·9*)* | *437 (42*·7*)* | *414 (40*·4*)* | *403 (39*·6*)* | *0*·003 |
| **Conscientiousness** _[7]_ *median (IQR)* | *16 (13-19)* | *16 (14-18)* | *16 (13-19)* | *16 (13-19)* | 0·996 |
| Bottom tertile | *1,003 (32*·7*)* | *302 (29*·5*)* | *347 (33*·8*)* | *354 (34*·8*)* |  |
| Middle tertile | *810 (26*·4*)* | *284 (27*·8*)* | *264 (25*·8*)* | *262 (25*·7*)* |  |
| Top tertile | *1,254 (40*·9*)* | *437 (42*·7*)* | *414 (40*·4*)* | *403 (40*·5*)* | 0·122 |
| **Extroversion** _[7]_ *median (IQR)* | *13 (11-15)* | *13 (11-15)* | *13 (11-15)* | *13 (11-16)* | 0·619 |
| Bottom tertile | *917 (29·9)* | *313 (30·6)* | *285 (27·8)* | *319 (31·3)* |  |
| Middle tertile | *1,097 (35·2)* | *362 (35·4)* | *391 (38·1)* | *326 (32·0)* |  |
| Top tertile | *1,071 (34·9)* | *348 (34·0)* | *349 (34·1)* | *374 (36·7)* | 0·055 |
| **Agreeableness** _[7]_ *median (IQR)* | *15 (12-17)* | *15 (12-17)* | *14 (12-17)* | *15 (12-17)* | 0·148 |
| Bottom tertile | *1,115 (36*·4*)* | *344 (33·6)* | *402 (39·2)* | *369 (36·2)* |  |
| Middle tertile | *1,025 (33*·4*)* | *370 (36·2)* | *327 (31·9)* | *328 (32·2)* |  |
| Top tertile | *927 (30*·2*)* | *309 (30·2)* | *296 (28·9)* | *322 (31·6)* | 0*·058* |
| **Openness** _[7]_ *median (IQR)* | *14 (11-17)* | *14 (12-17)* | *14 (11-16)* | *14 (11-17)* | 0·107 |
| Bottom tertile | *1,133 (37*·0*)* | *362 (35*·4*)* | *401 (39*·1*)* | *370 (36*·3*)* |  |
| Middle tertile | *896 (29*·2*)* | *300 (29*·3*)* | *307 (23*·0*)* | *289 (28*·4*)* |  |
| Top tertile | *1,038 (33*·8*)* | *361 (35*·3*)* | *317 (30*·9*)* | *360 (35*·3*)* | 0·170 |
| **Neuroticism** _[7]_ *median (IQR)* | *12 (9-14)* | *12 (9-14)* | *12 (9-14)* | *12 (9-14)* | 0·200 |
| Bottom tertile | *921 (30*·0*)* | *300 (29*·3*)* | *328 (32*·0*)* | *293 (28*·7*)* |  |
| Middle tertile | *543 (17*·7*)* | *198 (19*·4*)* | *168 (16*·4*)* | *177 (17*·4*)* |  |
| Top tertile | *1,603 (52*·3*)* | *525 (51*·3*)* | *529 (51*·6*)* | *549 (53*·9*)* | 0·244 |
| **COVID-19 infection (positive test)** | 217 (7·1) | 72 (7·0) | 58 (5·7) | 87 (8·5) | 0·040 |
| **Course of disease** |  |  |  |  |  |
| No symptoms | 44 (20·3) | 12 (16·7) | 9 (15·5) | 23 (26·4) |  |
| With symptoms at home | 152 (70·0) | 53 (73·6) | 42 (72·4) | 57 (65·5) |  |
| With symptoms at the hospital | 21 (9·7) | 7 (9·7) | 7 (12·1) | 7 (8·1) | 0·440 |
| Standard care | 15 (71·4) | 7 (100·0) | 3 (42·9) | 5 (71·4) |  |
| Intensive care | 6 (28·6) | 0 (0) | 4 (57·1) | 2 (28·6) | 0·061 |
| **Approval of the COVID-19 mitigation measures implemented by the government** | |  |  |  |  |
| No, they were unnecessary/  unjustified | 420 (13·7) | 129 (12·6) | 152 (14·8) | 139 (13·6) |  |
| Yes, partially | 1,077 (35·1) | 349 (34·1) | 352 (34·8) | 376 (36·9) |  |
| Yes, mainly or totally | 1,570 (51·2) | 545 (53·3) | 521 (50·8) | 504 (49·5) | 0·317 |
| **Vaccinated against COVID-19** |  |  |  |  |  |
| Fully immunized (two mRNA shots or one Johnson&Johnson) | 2,087 (68·1) | 651 (63·6) | 706 (68·9) | 730 (71·6) |  |
| Partially immunized (first mRNA shot) | 215 (7·0) | 52 (5·1) | 110 (10·7) | 53 (5·2) |  |
| Not yet, but made an appointment to get vaccinated | 178 (5·8) | 60 (5·9) | 65 (6·3) | 53 (5·2) |  |
| No, won´t get vaccinated | 587 (19·1) | 260 (25·4) | 144 (14·1) | 183 (18·0) | <0·001 |
| **Probability that friends/acquaintances are already vaccinated or will get vaccinated** | |  |  |  |  |
| Almost all or all are already vaccinated | 293 (9·6) | 72 (7·1) | 117 (11·4) | 104 (10·2) |  |
| Very likely | 1,011 (33·0) | 265 (25·9) | 407 (39·7) | 339 (33·3) |  |
| Rather likely | 712 (23·2) | 254 (24·8) | 233 (22·8) | 225 (22·1) |  |
| Nether likely nor unlikely | 817 (26·6) | 339 (33·1) | 196 (19·1) | 282 (27·7) |  |
| Unlikely | 115 (3·7) | 50 (4·9) | 33 (3·2) | 32 (3·1) |  |
| Very unlikely | 119 (3·9) | 43 (4·2) | 39 (3·8) | 37 (3·6) | <0·001 |
| **If not vaccinated yet, which percentage of the adult population would have to be vaccinated** **without showing any severe side-effects (death, disability, long-term disease) for you to change your mind and get vaccinated?** | |  |  |  |  |
| At least 50% | 27 (4·6) | 11 (4·3) | 7 (4·9) | 9 (4·9) |  |
| 51-60% | 25 (4·2) | 13 (5·0) | 4 (2·8) | 8 (4·4) |  |
| 61-70% | 28 (4·8) | 11 (4·2) | 10 (6·9) | 7 (3·8) |  |
| 71-80% | 58 (9·9) | 31 (11·9) | 11 (7·6) | 16 (8·7) |  |
| 81-90% | 45 (7·7) | 19 (7·3) | 10 (7·0) | 16 (8·7) |  |
| More than 90% | 92 (15·7) | 46 (17·7) | 17 (11·8) | 29 (15·9) |  |
| I won´t get vaccinated | 312 (53·1) | 129 (49·6) | 85 (59·0) | 98 (53·6) | <0·001 |
| **If not vaccinated yet, which conditions would increase your willingness to vaccinate** | |  |  |  |  |
| If the vaccine is free of charge | 80 (13·6) | 11 (7·6) | 40 (15·4) | 29 (15·8) | 0·054 |
| If I can pick the vaccine | 129 (22·0) | 25 (17·4) | 61 (23·5) | 43 (23·5) | 0·306 |
| If I get vaccinated at my work place | 18 (3·1) | 6 (4·2) | 5 (1·9) | 7 (3·8) | 0·353 |
| If I had the chance to win something (lottery) | 27 (4·6) | 3 (2·1) | 12 (4·6) | 12 (6·6) | 0·159 |
| If I would get a voucher | 49 (8·4) | 11 (7·6) | 22 (8·5) | 16 (8·7) | 0·934 |
| If I would receive a sticker saying that I got vaccinated | 7 (1·2) | 2 (1·4) | 2 (0·8) | 3 (1·6) | 0·686 |
| If I would get free food after getting the shot | 16 (2·7) | 5 (3·5) | 5 (1·9) | 6 (3·3) | 0·564 |
| **Willingness to get an annual COVID-19 booster, if necessary** _[8]_ |  |  |  |  |  |
| No, does not apply | 107 (4·3) | 42 (5·5) | 27 (3·1) | 38 (4·5) |  |
| No, does rather not apply | 329 (13·3) | 127 (16·6) | 86 (9·8) | 116 (13·9) |  |
| Yes, does rather apply | 890 (35·9) | 309 (40·5) | 297 (33·7) | 284 (34·0) |  |
| Yes, does apply | 1,154 (46·5) | 285 (37·4) | 471 (53·4) | 398 (47·6) | <0·001 |
| **BMI** [kg/m²] _[9]_ |  |  |  |  |  |
| Normal weight [BMI≥18·5 & <25] | 1,278 (45·2) | 446 (48·3) | 423 (44·4) | 409 (43·0) |  |
| Underweight [BMI<18·5] | 96 (3·4) | 30 (3·2) | 23 (2·4) | 43 (4·5) |  |
| Overweight [BMI≥25 & <30] | 905 (32·0) | 288 (31·2) | 317 (33·3) | 300 (31·5) |  |
| Obesity [BMI≥30] | 549 (19·4) | 160 (17·3) | 189 (19·9) | 200 (21·0) | 0·006 |
| **Frequency of physical activity done for at least 10 minutes which raises the heartbeat or the respiratory rate** |  |  |  |  |  |
| Less than once a week | 622 (20·3) | 155 (15·2) | 274 (26·7) | 193 (18·9) |  |
| 1-2 days a week | 802 (26·2) | 299 (29·2) | 256 (25·0) | 247 (24·3) |  |
| 3-4 days a week | 811 (26·4) | 278 (27·2) | 255 (24·9) | 278 (27·3) |  |
| 5-7 days a week | 832 (27·1) | 291 (28·4) | 240 (23·4) | 301 (29·5) | <0·001 |
| **Smoking status** |  |  |  |  |  |
| Never | 1,284 (41·9) | 442 (43·2) | 420 (41·0) | 422 (41·4) |  |
| Former | 839 (27·3) | 267 (26·1) | 264 (25·7) | 308 (30·2) |  |
| Current | 944 (30·8) | 314 (30·7) | 341 (33·3) | 289 (28·4) | 0·054 |
| **Chronic disease** _[10]_ | 1,243 (40·5) | 380 (37·2) | 463 (45·2) | 400 (39·3) | 0·001 |
| [1] P-values were calculated using Pearson's chi-squared test for categorical and k-sample equality-of-medians test for continuous variables because Shapiro-Wilk tests indicated that continuous variables were not normally distributed [2] household income tertiles were approximated because the distributions did not permit precise tertile cut-points [3] TKS-WLB^29^ [4] LOT-R^30^ [5] KUSIV3^31^ [6] questionnaire for empathy and perspective taking, German version^32^ [7] BFI-S^33^ [7] among participants who already got vaccinated or are planning to get vaccinated [8] 239 participants had missing information on BMI [10] asthma, COPD, chronical bronchitis, emphysema, heart attack, angina pectoris or coronary heart disease, cancer, hypertension, stroke or diabetes. | | | | | |

| **Supplementary table 2.**  Factors cross-sectionally associated with willingness to get an annual COVID-19 booster, if necessary, among participants who already got vaccinated or are planning to get vaccinated in Germany (n=880) | | | | | | | |
| --- | --- | --- | --- | --- | --- | --- | --- |
|  | No, (rather) not willing to get vaccinated every year (n=112) | Yes, rather willing to get vaccinated every year (n=297) | Yes, willing to get vaccinated every year (n=471) |  |  |  |  |
|  | n (%) | n (%) | n (%) | OR_crude_ (95% CI) | p-value | OR_adj_ (95% CI) ^[1]^ | p-value ^[1]^ |
| **Age (years)** |  |  |  |  |  |  |  |
| **18-25** | 30 (26·8) | 37 (12·5) | 18 (3·8) | Ref. |  | Ref. |  |
| **26-35** | 27 (24·1) | 55 (18·5) | 40 (8·5) | 1·88 (1·12-3·17) | 0·017 | 1·82 (1·03-3·21) | 0·038 |
| **36-45** | 18 (16·1) | 54 (18·2) | 65 (13·8) | 3·50 (2·09-5·88) | <0·001 | 2·64 (1·45-4·79) | 0·001 |
| **46-55** | 16 (14·3) | 52 (17·5) | 87 (18·5) | 4·91 (2·93-8·21) | <0·001 | 3·24 (1·78-5·90) | <0·001 |
| **56-65** | 15 (13·4) | 47 (15·8) | 125 (26·5) | 7·63 (4·57-12·7) | <0·001 | 4·22 (2·25-7·90) | <0·001 |
| **≥66** | 6 (5·3) | 52 (17·5) | 136 (28·9) | 9·40 (5·62-15·7) | <0·001 | 5·34 (2·54-11·2) | <0·001 |
| **Gender** |  |  |  |  |  |  |  |
| Women | 61 (54·5) | 151 (50·8) | 225 (47·8) | Ref. |  |  |  |
| Men | 51 (45·5) | 146 (49·2) | 246 (52·2) | 1·19 (0·92-1·54) | 0·182 |  |  |
| **Migration history** |  |  |  |  |  |  |  |
| First generation | 40 (35·7) | 116 (39·1) | 134 (28·5) | Ref. |  | Ref. |  |
| Second generation | 17 (15·2) | 22 (7·4) | 19 (4·0) | 0·49 (0·28-0·84) | 0·009 | 1·01 (0·55-1·84) | 0·980 |
| More than second generation/none | 55 (49·1) | 159 (53·5) | 318 (67·5) | 1·65 (1·25-2·18) | <0·001 | 1·39 (1·02-1·91) | 0·040 |
| **Ethnicity** |  |  |  |  |  |  |  |
| Other than White | 7 (6·3) | 23 (7·7) | 23 (4·9) | Ref. |  |  |  |
| White | 105 (93·7) | 274 (92·3) | 448 (95·1) | 1·40 (0·84-2·43) | 0·198 |  |  |
| **Educational attainment** |  |  |  |  |  |  |  |
| No university degree | 87 (77·7) | 205 (69·0) | 296 (62·8) | Ref. |  |  |  |
| University degree | 25 (22·3) | 92 (31·0) | 175 (37·2) | 1·53 (1·16-2·01) | 0·003 |  |  |
| **Household income** (tertiles defined at the regional level) _[2]_ |  |  |  |  |  |  |  |
| Approx. lowest tertile | 50 (44·6) | 114 (38·4) | 150 (31·9) | Ref. |  | Ref. |  |
| Approx. middle tertile | 19 (17·0) | 73 (24·6) | 122 (25·9) | 1·52 (1·09-2·13) | 0·014 | 1·65 (1·12-2·43) | 0·012 |
| Approx. highest tertile | 43 (38·4) | 110 (37·0) | 199 (42·2) | 1·42 (1·06-1·90) | 0·020 | 1·41 (0·98-2·03) | 0·064 |
| **Living area** |  |  |  |  |  |  |  |
| Urban | 83 (74·1) | 184 (61·9) | 305 (64·8) | Ref. |  |  |  |
| Rural | 29 (25·9) | 113 (38·1) | 166 (35·2) | 1·11 (0·85-1·45) | 0·437 |  |  |
| **Work status** |  |  |  |  |  |  |  |
| Full- (part-) time employed | 49 (43·8) | 124 (41·7) | 169 (35·9) | Ref. |  | Ref. |  |
| Full- (part-) time self-employed | 9 (8·0) | 18 (6·0) | 22 (4·6) | 0·81 (0·46-1·42) | 0·456 | 0·42 (0·22-0·79) | 0·008 |
| Unemployed | 7 (6·2) | 16 (5·4) | 16 (3·4) | 0·73 (0·39-1·35) | 0·310 | 1·25 (0·61-2·57) | 0·536 |
| Retired | 13 (11·6) | 73 (24·6) | 161 (34·2) | 2·00 (1·44-2·78) | <0·001 | 0·76 (0·45-1·30) | 0·322 |
| Student/in training/civil-/military-service | 15 (13·4) | 11 (3·7) | 13 (2·7) | 0·36 (0·19-0·68) | 0·002 | 1·05 (0·50-2·24) | 0·891 |
| Household | 5 (4·5) | 11 (3·7) | 31 (6·6) | 1·91 (1·01-3·59) | 0·045 | 1·81 (0·87-3·78) | 0·113 |
| Temporary contract | 3 (2·7) | 4 (1·4) | 6 (1·3) | 0·76 (0·26-2·22) | 0·613 | 0·63 (0·19-2·05) | 0·442 |
| Permanent contract | 11 (9·8) | 40 (13·5) | 53 (11·3) | 1·12 (0·74-1·70) | 0·587 | 1·32 (0·83-2·11) | 0·242 |
| **Satisfaction with work** |  |  |  |  |  |  |  |
| No, does not or does rather not apply | 50 (44·6) | 101 (34·0) | 112 (23·8) | Ref. |  | Ref. |  |
| Yes, does rather apply | 51 (45·5) | 160 (53·9) | 211 (44·8) | 1·43 (1·07-1·91) | 0·016 | 1·19 (0·85-1·66) | 0·307 |
| Yes, does totally apply | 11 (9·8) | 36 (12·1) | 148 (31·4) | 4·34 (2·90-6·48) | <0·001 | 3·03 (1·92-4·79) | <0·001 |
| **Work-Life balance** _[3]_ |  |  |  |  |  |  |  |
| Bottom tertile | 55 (49·1) | 111 (37·4) | 117 (24·8) | Ref. |  |  |  |
| Middle tertile | 36 (32·1) | 119 (40·1) | 135 (28·7) | 1·34 (0·98-1·82) | 0·063 |  |  |
| Top tertile | 21 (18·8) | 67 (22·5) | 219 (46·5) | 3·59 (2·58-5·01) | <0·001 |  |  |
| **Main job task** |  |  |  |  |  |  |  |
| Physical work with hands | 21 (18·7) | 48 (16·1) | 49 (10·4) | Ref. |  |  |  |
| Mental work with figures/symbols | 38 (33·9) | 89 (30·0) | 128 (27·2) | 1·37 (0·91-2·06) | 0·134 |  |  |
| Contact/Communication with other people | 21 (18·8) | 54 (18·2) | 84 (17·8) | 1·53 (0·98-2·41) | 0·062 |  |  |
| Not working | 32 (28·6) | 106 (35·7) | 210 (44·6) | 2·12 (1·42-3·15) | <0·001 |  |  |
| **Political preference/involvement** (last elections) |  |  |  |  |  |  |  |
| Did not vote | 27 (24·1) | 55 (18·5) | 57 (12·1) | Ref. |  |  |  |
| Opposition parties | 39 (34·8) | 87 (29·3) | 156 (33·1) | 1·73 (1·17-2·54) | 0·006 |  |  |
| Governing parties | 46 (41·1) | 155 (52·2) | 258 (54·8,) | 1·90 (1·33-2·72) | <0·001 |  |  |
| **Participation at religious meetings** |  |  |  |  |  |  |  |
| At least once a month | 39 (34·8) | 56 (18·8) | 47 (10·0) | Ref. |  | Ref. |  |
| Less than once a month | 12 (10·7) | 40 (13·5) | 55 (11·7) | 2·39 (1·48-3·85) | <0·001 | 1·58 (0·92-2·71) | 0·096 |
| Never, or almost never | 61 (54·5) | 201 (67·7) | 369 (78·3) | 3·10 (2·19-4·41) | <0·001 | 1·72 (1·13-2·62) | 0·011 |
| **Contact with a close person (except children) I can talk to** |  |  |  |  |  |  |  |
| Less than once a week | 34 (30·4) | 45 (15·2) | 32 (6·8) | Ref. |  | Ref. |  |
| At least once a week | 22 (19·6) | 64 (21·6) | 78 (16·5) | 2·47 (1·57-3·90) | <0·001 | 1·32 (0·78-2·21) | 0·297 |
| Daily | 56 (50·0) | 188 (63·3) | 361 (76·7) | 3·96 (2·68-5·85) | <0·001 | 1·57 (1·00-2·47) | 0·050 |
| **In conversations I consider myself a:** |  |  |  |  |  |  |  |
| *“No, but…” type* | 33 (29·5) | 81 (27·3) | 133 (28·2) | Ref. |  |  |  |
| *“Yes, and…” type* | 79 (70·5) | 216 (72·7) | 338 (71·8) | 1·00 (0·75-1·33) | 0·997 |  |  |
| **Optimism** _[4]_ |  |  |  |  |  |  |  |
| Bottom tertile | 68 (60·7) | 130 (43·8) | 185 (39·3) | Ref. |  |  |  |
| Middle tertile | 27 (24·1) | 104 (35·0) | 115 (24·4) | 1·08 (0·80-1·46) | 0·608 |  |  |
| Top tertile | 17 (15·2) | 63 (21·2) | 171 (36·3) | 2·43 (1·76-3·37) | <0·001 |  |  |
| **Interpersonal trust** _[5]_ |  |  |  |  |  |  |  |
| Bottom tertile | 35 (31·3) | 64 (21·6) | 91 (19·3) | Ref. |  |  |  |
| Middle tertile | 54 (48·2) | 153 (51·5) | 180 (38·2) | 1·04 (0·75-1·45) | 0·796 |  |  |
| Top tertile | 23 (20·5) | 80 (26·9) | 200 (42·5) | 2·26 (1·58-3·24) | <0·001 |  |  |
| **Empathy** _[6]_ |  |  |  |  |  |  |  |
| Bottom tertile | 49 (43·8) | 111 (37·4) | 133 (28·2) | Ref. |  |  |  |
| Middle tertile | 35 (31·2) | 102 (34·3) | 139 (29·5) | 1·25 (0·92-1·71) | 0·154 |  |  |
| Top tertile | 28 (25·0) | 84 (28·3) | 199 (42·3) | 2·13 (1·56-2·92) | <0·001 |  |  |
| **Perspective taking** _[6]_ |  |  |  |  |  |  |  |
| Bottom tertile | 41 (36·6) | 109 (36·7) | 140 (29·7) | Ref. |  |  |  |
| Middle tertile | 30 (26·8) | 86 (29·0) | 118 (25·1) | 1·09 (0·79-1·52) | 0·589 |  |  |
| Top tertile | 41 (36·6) | 102 (34·3) | 213 (45·2) | 1·53 (1·13-2·07) | 0·006 |  |  |
| **Conscientiousness** _[7]_ |  |  |  |  |  |  |  |
| Bottom tertile | 66 (58·9) | 130 (43·8) | 121 (25·7) | Ref. |  |  |  |
| Middle tertile | 22 (19·7) | 88 (29·6) | 133 (28·2) | 2·08 (1·51-2·86) | <0·001 |  |  |
| Top tertile | 24 (21·4) | 79 (26·6) | 217 (46·1) | 3·45 (2·52-4·72) | <0·001 |  |  |
| **Extroversion** _[7]_ |  |  |  |  |  |  |  |
| Bottom tertile | 31 (27·7) | 84 (28·3) | 130 (27·6) | Ref. |  |  |  |
| Middle tertile | 45 (40·2) | 130 (43·8) | 159 (33·8) | 0·83 (0·61-1·14) | 0·250 |  |  |
| Top tertile | 36 (31·1) | 83 (27·9) | 182 (38·6) | 1·30 (0·94-1·82) | 0·117 |  |  |
| **Agreeableness** _[7]_ |  |  |  |  |  |  |  |
| Bottom tertile | 58 (51·8) | 139 (46·8) | 150 (31·8) | Ref. |  |  |  |
| Middle tertile | 37 (33·0) | 94 (31·7) | 153 (32·5) | 1·49 (1·10-2·01) | 0·009 |  |  |
| Top tertile | 17 (15·2) | 64 (21·5) | 168 (35·7) | 2·70 (1·94-3·76) | <0·001 |  |  |
| **Openness** _[7]_ |  |  |  |  |  |  |  |
| Bottom tertile | 39 (34·8) | 133 (44·8) | 178 (37·8) | Ref. |  |  |  |
| Middle tertile | 44 (39·3) | 93 (31·3) | 133 (28·2) | 0·87 (0·64-1·17) | 0·356 |  |  |
| Top tertile | 29 (25·9) | 71 (23·9) | 160 (34·0) | 1·43 (1·05-1·97) | 0·025 |  |  |
| **Neuroticism** _[7]_ |  |  |  |  |  |  |  |
| Bottom tertile | 22 (19·6) | 80 (26·9) | 180 (38·2) | Ref. |  |  |  |
| Middle tertile | 22 (19·6) | 53 (17·9) | 75 (15·9) | 0·55 (0·38-0·82) | 0·003 |  |  |
| Top tertile | 68 (60·8) | 164 (55·2) | 216 (45·9) | 0·52 (0·39-0·70) | <0·001 |  |  |
| **COVID-19 infection (positive test)** | 10 (8·9) | 18 (6·1) | 26 (5·5) |  |  |  |  |
| **Approval of the COVID-19 measures implemented by the government** |  |  |  | 0·74 (0·44-1·26) | 0·274 |  |  |
| No, they were unnecessary/ unjustified | 23 (20·5) | 36 (12·1) | 23 (4·9) | Ref. |  | Ref. |  |
| Yes, partially | 58 (51·8) | 131 (44·1) | 114 (24·2) | 1·60 (1·01-2·52) | 0·045 | 1·71 (1·05-2·81) | 0·032 |
| Yes, mainly or totally | 31 (27·7) | 130 (43·8) | 334 (70·9) | 5·51 (3·50-8·67) | <0·001 | 4·25 (2·60-6·96) | <0·001 |
| **Probability that friends/acquaintances are already vaccinated or will get vaccinated** |  |  |  |  |  |  |  |
| Almost all or all are already ··vaccinated | 13 (11·6) | 24 (8·1) | 65 (13·8) | Ref. |  | Ref. |  |
| Very likely | 27 (24·1) | 101 (34·0) | 266 (56·5) | 1·28 (0·81-2·01) | 0·291 | 1·29 (0·78-2·11) | 0·319 |
| Rather likely | 34 (30·4) | 99 (33·3) | 89 (18·9) | 0·44 (0·27-0·70) | 0·001 | 0·61 (0·36-1·02) | 0·060 |
| Nether likely nor unlikely | 31 (27·7) | 65 (21·9) | 46 (9·8) | 0·31 (0·18-0·51) | <0·001 | 0·49 (0·28-0·86) | 0·013 |
| Unlikely | 5 (4·4) | 6 (2·0) | 2 (0·4) | 0·13 (0·05-0·30) | <0·001 | 0·39 (0·12-1·27) | 0·117 |
| Very unlikely | 2 (1·8) | 2 (0·7) | 3 (0·6) | 0·35 (0·08-1·60) | 0·176 | 0·89 (0·15-5·54) | 0·893 |
| **BMI** [kg/m²] |  |  |  |  |  |  |  |
| Normal weight [BMI≥18·5 & <25] | 53 (51·0) | 132 (48·0) | 162 (37·0) | Ref. |  | Ref. |  |
| Underweight [BMI<18·5] | 4 (3·9) | 7 (2·6) | 6 (1·4) | 0·61 (0·25-1·51) | 0·287 | 0·48 (0·18-1·26) | 0·136 |
| Overweight [BMI≥25 & <30] | 24 (23·0) | 99 (36·0) | 156 (35·6) | 1·50 (1·11-2·03) | 0·008 | 1·23 (0·87-1·75) | 0·236 |
| Obesity [BMI≥30] | 23 (22·1) | 37 (13·4) | 114 (26·0) | 1·98 (1·37-2·87) | <0·001 | 1·63 (1·07-2·49) | 0·022 |
| **Frequency of physical activity done for at least 10 minutes which raises the heartbeat or the respiratory rate** |  |  |  |  |  |  |  |
| Less than once a week | 25 (22·3) | 61 (20·5) | 143 (30·3) | Ref. |  |  |  |
| 1-2 days a week | 29 (25·9) | 91 (30·6) | 102 (21·7) | 0·56 (0·39-0·80) | 0·002 |  |  |
| 3-4 days a week | 36 (32·2) | 83 (28·0) | 105 (22·3) | 0·54 (0·38-0·78) | 0·001 |  |  |
| 5-7 days a week | 22 (19·6) | 62 (20·9) | 121 (25·7) | 0·89 (0·61-1·29) | 0·529 |  |  |
| **Smoking status** |  |  |  |  |  |  |  |
| Never | 51 (45·5) | 124 (41·8) | 184 (39·1) | Ref. |  |  |  |
| Former | 27 (24·1) | 74 (24·9) | 137 (29·1) | 1·29 (0·94-1·78) | 0·114 |  |  |
| Current | 34 (30·4) | 99 (33·3) | 150 (31·8) | 1·10 (0·82-1·48) | 0·536 |  |  |
| **Chronic disease** _[8]_ | 45 (40·2) | 114 (38·4) | 260 (55·2) | 1·83 (1·41-2·37) | <0·001 |  |  |
| [1] mutually adjusted for all variables for which adjusted odds ratios with 95% confidence intervals and adjusted p-values are reported [2] household income tertiles were approximated because the distributions did not permit precise tertile cut-points [3] TKS-WLB^29^ [4] LOT-R^30^ [5] KUSIV3^31^ [6] questionnaire for empathy and perspective taking, German version^32^ [7] BFI-S^33^ [8] asthma, COPD, chronical bronchitis, emphysema, heart attack, angina pectoris or coronary heart disease, cancer, hypertension, stroke or diabetes | | | | | | | |

| **Supplementary table 3** Factors cross-sectionally associated with willingness to get an annual COVID-19 booster, if necessary, among participants who already got vaccinated or are planning to get vaccinated in Austria (N=836) | | | | | | | |
| --- | --- | --- | --- | --- | --- | --- | --- |
|  | No, (rather) not willing to get vaccinated every year (n=154) | Yes, rather willing to get vaccinated every year (n=284) | Yes, willing to get vaccinated every year (n=398) |  |  |  |  |
|  | n (%) | n (%) | n (%) | OR_crude_ (95% CI) | p-value | OR_adj_ (95% CI) ^[1]^ | p-value ^[1]^ |
| **Age (years)** |  |  |  |  |  |  |  |
| **18-25** | 28 (18·1) | 45 (15·9) | 28 (7·0) | Ref. |  | Ref. |  |
| **26-35** | 26 (16·9) | 47 (16·5) | 48 (12·1) | 1·54 (0·95-2·50) | 0·080 | 3·26 (1·65-5·83) | <0·001 |
| **36-45** | 36 (23·4) | 60 (21·1) | 54 (13·6) | 1·33 (0·84-2·10) | 0·228 | 2·54 (1·34-4·65) | 0·004 |
| **46-55** | 32 (20·8) | 51 (18·0) | 75 (18·8) | 1·97 (1·24-3·13) | 0·004 | 3·06 (1·62-5·78) | 0·001 |
| **56-65** | 24 (15·6) | 48 (16·9) | 99 (24·9) | 3·07 (1·93-4·89) | <0·001 | 4·12 (2·07-8·19) | <0·001 |
| **≥66** | 8 (5·2) | 33 (11·6) | 94 (23·6) | 5·42 (3·25-9·03) | <0·001 | 4·09 (1·81-8·26) | 0·001 |
| **Gender** |  |  |  |  |  |  |  |
| Women | 88 (57·1) | 154 (54·2) | 167 (42·0) | Ref. |  | Ref. |  |
| Men | 66 (42·9) | 130 (45·8) | 231 (58·0) | 1·65 (1·27-2·13) | <0·001 | 1·50 (1·11-2·03) | 0·008 |
| **Migration history** |  |  |  |  |  |  |  |
| First generation | 35 (22·7) | 72 (25·4) | 77 (19·4) | Ref. |  |  |  |
| Second generation | 19 (12·3) | 23 (8·1) | 31 (7·8) | 0·88 (0·53-1·47) | 0·630 |  |  |
| More than second generation/none | 100 (65·0) | 189 (66·5) | 290 (72·8) | 1·30 (0·96-1·77) | 0·091 |  |  |
| **Ethnicity** |  |  |  |  |  |  |  |
| Other than White | 12 (7·8) | 343 (12·0) | 29 (7·3) |  |  |  |  |
| White | 142 (92·2) | 250 (88·0) | 369 (92·7) | Ref. |  |  |  |
| **Educational attainment** |  |  |  | 1·24 (0·81-1·91) | 0·318 |  |  |
| No university degree | 121 (78·6) | 230 (81·0) | 315 (79·2) | Ref. |  |  |  |
| University degree | 33 (21·4) | 54 (19·0) | 83 (20·8) | 1·02 (0·74-1·42) | 0·896 |  |  |
| **Household income** (tertiles defined at the regional level) _[2]_ |  |  |  |  |  |  |  |
| Approx. lowest tertile | 57 (37·0) | 109 (38·4) | 148 (37·2) |  |  |  |  |
| Approx. middle tertile | 34 (22·1) | 71 (25·0) | 89 (22·4) | Ref. |  |  |  |
| Approx. highest tertile | 63 (40·9) | 104 (36·6) | 161 (40·5) | 0·98 (0·70-1·36) | 0·888 |  |  |
| **Living area** |  |  |  | 1·04 (0·77-1·39) | 0·803 |  |  |
| Urban | 95 (61·7) | 161 (56·7) | 215 (54·0) | Ref. |  |  |  |
| Rural | 59 (38·3) | 123 (43·3) | 183 (46·0) | 1·23 (0·95-1·59) | 0·120 |  |  |
| **Work status** |  |  |  |  |  |  |  |
| Full- (part-) time employed | 78 (50·7) | 113 (39·8) | 119 (29·9) | Ref. |  | Ref. |  |
| Full- (part-) time self-employed | 11 (7·1) | 16 (5·6) | 24 (6·0) | 1·37 (0·78-2·41) | 0·271 | 0·91 (0·48-1·71) | 0·762 |
| Unemployed | 12 (7·8) | 15 (5·3) | 24 (6·0) | 1·32 (0·75-2·32) | 0·338 | 1·33 (0·71-2·51) | 0·378 |
| Retired | 22 (14·3) | 57 (20·1) | 151 (37·9) | 3·15 (2·24-4·43) | <0·001 | 2·03 (1·17-3·51) | 0·008 |
| Student/in training/civil-/military-service | 10 (6·5) | 29 (10·2) | 28 (7·0) | 1·34 (0·83-2·18) | 0·232 | 3·51(1·77-6·97) | <0·001 |
| Household | 2 (1·3) | 9 (3·2) | 9 (2·3) | 1·60 (0·70-3·65) | 0·265 | 2·60 (1·02-6·62) | 0·046 |
| Temporary contract | 1 (0·6) | 7 (2·4) | 3 (0·8) | 1·05 (0·38-2·92) | 0·929 | 1·48 (0·49-4·52) | 0·500 |
| Permanent contract | 18 (11·7) | 38 (13·4) | 40 (10·1) | 1·25 (0·82-1·90) | 0·310 | 1·23 (0·78-1·95) | 0·375 |
| **Satisfaction with work** |  |  |  |  |  |  |  |
| No, does not or does rather not apply | 55 (35·7) | 81 (28·5) | 99 (24·9) | Ref. |  | Ref. |  |
| Yes, does rather apply | 74 (48·1) | 159 (56·0) | 165 (41·5) | 1·08 (0·80-1·46) | 0·610 | 0·92 (0·65-1·29) | 0·612 |
| Yes, does totally apply | 25 (16·2) | 44 (15·5) | 134 (33·7) | 2·66 (1·83-3·88) | <0·001 | 1·67 (1·09-2·55) | 0·019 |
| **Work-Life balance** _[3]_ |  |  |  |  |  |  |  |
| Bottom tertile | 68 (44·2) | 114 (40·2) | 115 (28·9) | Ref. |  | Ref. |  |
| Middle tertile | 43 (27·9) | 94 (33·1) | 99 (24·9) | 1·20 (0·87-1·64) | 0·266 |  |  |
| Top tertile | 43 (27·9) | 76 (26·7) | 184 (46·2) | 2·30 (1·68-3·13) | <0·001 |  |  |
| **Main job task** |  |  |  |  |  |  |  |
| Physical work with hands | 31 (20·1) | 53 (18·7) | 46 (11·6) | Ref. |  |  |  |
| Mental work with figures/symbols | 44 (28·6) | 80 (28·2) | 84 (21·1) | 1·20 (0·80-1·80) | 0·368 |  |  |
| Contact/Communication with other people | 39 (25·3) | 66 (23·2) | 75 (18·8) | 1·23 (0·81-1·87) | 0·324 |  |  |
| Not working | 40 (26·0) | 85 (29·9) | 193 (48·5) | 2·62 (1·78-3·85) | <0·001 |  |  |
| **Political preference/involvement** (last elections) |  |  |  |  |  |  |  |
| Did not vote | 39 (25·3) | 51 (18·0) | 39 (9·8) | Ref. |  | Ref. |  |
| Opposition parties | 52 (33·8) | 89 (31·3) | 131 (32·9) | 2·03 (1·37-3·00) | <0·001 | 1·91 (1·23-2·98) | 0·004 |
| Governing parties | 63 (40·9) | 144 (50·7) | 228 (57·3) | 2·51 (1·74-3·62) | <0·001 | 2·21 (1·47-3·33) | <0·001 |
| **Participation at religious meetings** |  |  |  |  |  |  |  |
| At least once a month | 27 (17·6) | 34 (12·0) | 48 (12·1) | Ref. |  |  |  |
| Less than once a month | 25 (16·2) | 61 (21·5) | 65 (16·3) | 1·14 (0·72-1·81) | 0·570 |  |  |
| Never, or almost never | 102 (66·2) | 189 (66·5) | 285 (71·6) | 1·34 (0·91-1·98) | 0·137 |  |  |
| **Contact with a close person (except children) I can talk to** |  |  |  |  |  |  |  |
| Less than once a week | 19 (12·3) | 24 (8·4) | 19 (4·8) | Ref. |  |  |  |
| At least once a week | 30 (19·5) | 44 (15·5) | 64 (16·1) | 1·82 (1·05-3·18) | 0·034 |  |  |
| Daily | 154 (68·2) | 216 (76·1) | 315 (79·1) | 2·21 (1·36-3·57) | 0·001 |  |  |
| **In conversations I consider myself a:** |  |  |  |  |  |  |  |
| *“No, but…” type* | 46 (29·9) | 79 (27·8) | 91 (22·9) | Ref. |  |  |  |
| *“Yes, and…” type* | 108 (70·1) | 205 (72·2) | 307 (77·1) | 1·33 (0·99-1·77) | 0·055 |  |  |
| **Optimism** _[4]_ |  |  |  |  |  |  |  |
| Bottom tertile | 62 (40·2) | 106 (37·3) | 122 (30·6) | Ref. |  |  |  |
| Middle tertile | 40 (26·0) | 76 (26·8) | 95 (23·9) | 1·14 (0·82-1·58) | 0·446 |  |  |
| Top tertile | 52 (33·8) | 102 (35·9) | 181 (45·5) | 1·58 (1·17-2·13) | 0·003 |  |  |
| **Interpersonal trust** _[5]_ |  |  |  |  |  |  |  |
| Bottom tertile | 39 (25·3) | 62 (21·8) | 87 (21·9) | Ref. |  |  |  |
| Middle tertile | 72 (46·8) | 134 (47·2) | 164 (41·2) | 0·97 (0·70-1·35) | 0·858 |  |  |
| Top tertile | 43 (27·9) | 88 (31·0) | 147 (36·9) | 1·34 (0·94-1·91) | 0·102 |  |  |
| **Empathy** _[6]_ |  |  |  |  |  |  |  |
| Bottom tertile | 64 (41·6) | 95 (33·5) | 120 (30·2) | Ref. |  |  |  |
| Middle tertile | 34 (22·1) | 79 (27·8) | 113 (28·4) | 1·42 (1·02-1·97) | 0·039 |  |  |
| Top tertile | 56 (36·3) | 110 (38·7) | 165 (41·4) | 1·36 (1·01-1·84) | 0·043 |  |  |
| **Perspective taking** _[6]_ |  |  |  |  |  |  |  |
| Bottom tertile | 52 (33·8) | 111 (39·1) | 123 (30·9) | Ref. |  |  |  |
| Middle tertile | 45 (29·2) | 57 (20·1) | 114 (28·6) | 1·27 (0·90-1·77) | 0·170 |  |  |
| Top tertile | 57 (37·0) | 116 (40·8) | 161 (40·5) | 1·18 (0·88-1·58) | 0·267 |  |  |
| **Conscientiousness** _[7]_ |  |  |  |  |  |  |  |
| Bottom tertile | 58 (37·7) | 118 (41·5) | 116 (29·1) | Ref. |  |  |  |
| Middle tertile | 34 (22·1) | 90 (31·7) | 120 (30·2) | 1·45 (1·06-1·99) | 0·021 |  |  |
| Top tertile | 62 (40·2) | 76 (26·8) | 162 (40·7) | 1·49 (1·10-2·02) | 0·011 |  |  |
| **Extroversion** _[7]_ |  |  |  |  |  |  |  |
| Bottom tertile | 46 (29·9) | 85 (29·9) | 133 (33·4) | Ref. |  |  |  |
| Middle tertile | 56 (36·3) | 104 (36·6) | 113 (28·4) | 0·73 (0·53-1·00) | 0·053 |  |  |
| Top tertile | 52 (33·8) | 95 (33·5) | 152 (38·2) | 1·01 (0·74-1·39) | 0·929 |  |  |
| **Agreeableness** _[7]_ |  |  |  |  |  |  |  |
| Bottom tertile | 68 (44·2) | 111 (39·1) | 116 (29·2) | Ref. |  |  |  |
| Middle tertile | 49 (31·8) | 93 (32·7) | 141 (35·4) | 1·50 (1·10-2·04) | 0·009 |  |  |
| Top tertile | 37 (24·0) | 80 (28·2) | 141 (35·4) | 1·83 (1·34-2·52) | <0·001 |  |  |
| **Openness** _[7]_ |  |  |  |  |  |  |  |
| Bottom tertile | 66 (42·8) | 104 (36·6) | 134 (33·7) | Ref. |  |  |  |
| Middle tertile | 42 (27·3) | 91 (32·1) | 109 (27·4) | 1·12 (0·82-1·53) | 0·492 |  |  |
| Top tertile | 46 (29·9) | 89 (31·3) | 155 (38·9) | 1·47 (1·08-2·00) | 0·014 |  |  |
| **Neuroticism** _[7]_ |  |  |  |  |  |  |  |
| Bottom tertile | 44 (28·6) | 59 (20·8) | 127 (31·9) | Ref. |  |  |  |
| Middle tertile | 27 (17·5) | 54 (19·0) | 71 (17·8) | 0·79 (0·53-1·17) | 0·240 |  |  |
| Top tertile | 83 (53·9) | 171 (60·2) | 200 (50·3) | 0·73 (0·54-0·99) | 0·041 |  |  |
| **COVID-19 infection (positive test)** | 21 (13·6) | 27 (9·5) | 22 (5·5) | 0·48 (0·31-0·76) | 0·002 |  |  |
| **Approval of the COVID-19 measures implemented by the government** |  |  |  |  |  |  |  |
| No, they were unnecessary/ unjustified | 37 (24·0) | 12 (4·2) | 14 (3·5) | Ref. |  | Ref. |  |
| Yes, partially | 80 (52·0) | 140 (49·3) | 82 (20·6) | 2·94 (1·69-5·09) | <0·001 | 3·74 (2·02-6·91) | <0·001 |
| Yes, mainly or totally | 37 (24·0) | 132 (46·5) | 302 (75·9) | 13·1 (7·50-22·9) | <0·001 | 13·2 (7·11-24·5) | <0·001 |
| **Probability that friends/acquaintances are already vaccinated or will get vaccinated** |  |  |  |  |  |  |  |
| Almost all or all are already ··vaccinated | 6 (3·9) | 19 (6·7) | 55 (13·8) | Ref. |  | Ref. |  |
| Very likely | 35 (22·7) | 96 (33·8) | 192 (48·2) | 0·67 (0·40-1·12) | 0·123 | 0·50 (0·28-0·87) | 0·015 |
| Rather likely | 38 (24·7) | 86 (30·3) | 81 (20·4) | 0·32 (0·19-0·54) | <0·001 | 0·28 (0·16-0·50) | <0·001 |
| Nether likely nor unlikely | 67 (43·5) | 73 (25·7) | 63 (15·8) | 0·18 (0·11-0·32) | <0·001 | 0·17 (0·09-0·30) | <0·001 |
| Unlikely | 6 (3·9) | 8 (2·8) | 3 (0·8) | 0·13 (0·05-0·34) | <0·001 | 0·14 (0·05-0·44) | 0·001 |
| Very unlikely | 2 (1·3) | 2 (0·7) | 4 (1·0) | 0·38 (0·09-1·58) | 0·182 | 0·65 (0·13-3·23) | 0·601 |
| **BMI** [kg/m²] |  |  |  |  |  |  |  |
| Normal weight [BMI≥18·5 & <25] | 71 (49·0) | 127 (47·9) | 139 (36·4) | Ref. |  |  |  |
| Underweight [BMI<18·5] | 7 (4·8) | 15 (5·7) | 10 (2·6) | 0·77 (0·40-1·47) | 0·421 |  |  |
| Overweight [BMI≥25 & <30] | 37 (25·5) | 81 (30·6) | 132 (34·6) | 1·57 (1·15-2·14) | 0·004 |  |  |
| Obesity [BMI≥30] | 30 (20·7) | 42 (15·8) | 101 (26·4) | 1·80 (1·26-2·58) | 0·001 |  |  |
| **Frequency of physical activity done for at least 10 minutes which raises the heartbeat or the respiratory rate** |  |  |  |  |  |  |  |
| Less than once a week | 25 (16·2) | 47 (16·6) | 74 (18·6) | Ref. |  |  |  |
| 1-2 days a week | 44 (28·6) | 79 (27·8) | 88 (22·1) | 0·72 (0·48-1·07) | 0·108 |  |  |
| 3-4 days a week | 41 (26·6) | 83 (29·2) | 111 (27·9) | 0·90 (0·61-1·33) | 0·590 |  |  |
| 5-7 days a week | 44 (28·6) | 75 (26·4) | 125 (31·4) | 1·00 (0·68-1·48) | 1.000 |  |  |
| **Smoking status** |  |  |  |  |  |  |  |
| Never | 61 (39·6) | 118 (41·5) | 163 (41·0) | Ref. |  |  |  |
| Former | 43 (27·9) | 84 (29·6) | 137 (34·4) | 1·17 (0·86-1·58) | 0·322 |  |  |
| Current | 50 (32·5) | 82 (28·9) | 98 (24·6) | 0·81 (0·59-1·10) | 0·175 |  |  |
| **Chronic disease** _[8]_ | 48 (31·2) | 103 (36·3) | 204 (51·3,) | 1·95 (1·50-2·54 ) | <0·001 |  |  |
| [1] mutually adjusted for all variables for which adjusted odds ratios with 95% confidence intervals and adjusted p-values are reported [2] household income tertiles were approximated because the distributions did not permit precise tertile cut-points [3] TKS-WLB^29^ [4] LOT-R^30^ [5] KUSIV3^31^ [6] questionnaire for empathy and perspective taking, German version^32^ [7] BFI-S^33^ [8] asthma, COPD, chronical bronchitis, emphysema, heart attack, angina pectoris or coronary heart disease, cancer, hypertension, stroke or diabetes | | | | | | | |

| **Supplementary table 4** Factors cross-sectionally associated with willingness to get an annual COVID-19 booster, if necessary, among participants who already got vaccinated or are planning to get vaccinated in Switzerland (N=763) | | | | | | | |
| --- | --- | --- | --- | --- | --- | --- | --- |
|  | No, (rather) not willing to get vaccinated every year (n=169) | Yes, rather willing to get vaccinated every year (n=309) | Yes, willing to get vaccinated every year (n=285) |  |  |  |  |
|  | n (%) | n (%) | n (%) | OR_crude_ (95% CI) | p-value | OR_adj_ (95% CI) ^[1]^ | p-value ^[1]^ |
| **Age (years)** |  |  |  |  |  |  |  |
| **18-25** | 23 (13·6) | 38 (12·3) | 21 (7·3) | Ref. |  |  |  |
| **26-35** | 33 (19·5) | 60 (19·4) | 24 (8·4) | 0·88 (0·53-1·48) | 0·640 |  |  |
| **36-45** | 45 (26·6) | 61 (19·8) | 29 (10·2) | 0·79 (0·48-1·31) | 0·366 |  |  |
| **46-55** | 28 (16·6) | 52 (16·8) | 43 (15·1) | 1·45 (0·86-2·43) | 0·163 |  |  |
| **56-65** | 26 (15·4) | 56 (18·1) | 78 (27·4) | 2·50 (1·51-4·14) | <0·001 |  |  |
| **≥66** | 14 (8·3) | 42 (13·6) | 90 (31·6) | 4·39 (2·60-7·40) | <0·001 |  |  |
| **Gender** |  |  |  |  |  |  |  |
| Women | 94 (55·6) | 145 (46·9) | 127 (44·6) | Ref. |  |  |  |
| Men | 75 (44·4) | 164 (53·1) | 158 (55·4) | 1·33 (1·02-1·73) | 0·035 |  |  |
| **Migration history** |  |  |  |  |  |  |  |
| First generation | 40 (23·7) | 92 (29·8) | 69 (24·2) | Ref. |  |  |  |
| Second generation | 25 (14·8) | 51 (16·5) | 27 (9·5) | 0·74 (0·48-1·14) | 0·179 |  |  |
| More than second generation/none | 104 (61·5) | 166 (53·7) | 189 (66·3) | 1·13 (0·84-1·54) | 0·419 |  |  |
| **Ethnicity** |  |  |  |  |  |  |  |
| Other than White | 22 (13·0) | 31 (10·0) | 18 (6·3) | Ref. |  |  |  |
| White | 147 (87·0) | 278 (90·0) | 267 (93·7) | 1·75 (1·11-2·74) | 0·179 |  |  |
| **Educational attainment** |  |  |  | 1·13 (0·84-1·54) | 0·419 |  |  |
| No university degree | 144 (85·2) | 240 (77·7) | 225 (78·9) | Ref. |  |  |  |
| University degree | 25 (14·8) | 69 (22·3) | 60 (21·1) | 1·23 (0·89-1·71) | 0·208 |  |  |
| **Household income** (tertiles defined at the regional level) _[2]_ |  |  |  |  |  |  |  |
| Aprrox. lowest tertile | 48 (28·4) | 105 (34·0) | 86 (30·1) | Ref. |  |  |  |
| Approx. middle tertile | 53 (31·4) | 90 (29·1) | 88 (30·9) | 1·00 (0·71-1·39) | 0·977 |  |  |
| Approx. highest tertile | 68 (40·2) | 114 (36·9) | 111 (39·0) | 0·98 (0·72-1·35) | 0·919 |  |  |
| **Living area** |  |  |  |  |  |  |  |
| Urban | 66 (39·0) | 144 (46·6) | 135 (47·4) | Ref. |  |  |  |
| Rural | 103 (61·0) | 165 (53·4) | 150 (52·6) | 0·81 (0·62-1·06) | 0·128 |  |  |
| **Work status** |  |  |  |  |  |  |  |
| Full- (part-) time employed | 86 (50·9) | 134 (43·4) | 92 (32·3) | Ref. |  | Ref. |  |
| Full- (part-) time self-employed | 12 (7·1) | 16 (5·2) | 21 (7·4) | 1·56 (0·87-2·78) | 0·133 | 1·19 (0·65-2·18) | 0·577 |
| Unemployed | 5 (3·0) | 10 (3·2) | 12 (4·2) | 1·86 (0·88-3·93) | 0·103 | 2·57 (1·15-5·79) | 0·022 |
| Retired | 15 (8·9) | 56 (18·1) | 106 (37·2) | 3·71 (2·57-5·34) | <0·001 | 2·32 (1·55-3·47) | <0·001 |
| Student/in training/civil-/military-service | 9 (5·3) | 24 (7·8) | 13 (4·6) | 1·17 (0·67-2·04) | 0·586 | 1·16 (0·63-2·13) | 0·626 |
| Household | 8 (4·7) | 20 (6·5) | 15 (5·2) | 1·17 (0·67-2·05) | 0·246 | 1·51 (0·81-2·80) | 0·194 |
| Temporary contract | 0 (0·0) | 13 (4·2) | 2 (0·7) | 1·42 (0·79-2·55) | 0·634 | 2·19 (0·87-5·54) | 0·097 |
| Permanent contract | 34 (20·1) | 36 (11·6) | 24 (8·4) | 1·23 (0·53-2·88) | 0·147 | 0·90 (0·57-1·41) | 0·634 |
| **Satisfaction with work** |  |  |  |  |  |  |  |
| No, does not or does rather not apply | 44 (26·0) | 52 (16·8) | 33 (11·6) | Ref. |  | Ref. |  |
| Yes, does rather apply | 95 (56·2) | 174 (56·3) | 132 (46·3) | 1·55 (1·07-2·25) | 0·020 | 1·38 (0·91-2·08) | 0·128 |
| Yes, does totally apply | 30 (17·8) | 83 (26·9) | 120 (42·1) | 3·31 (2·19-5·00) | <0·001 | 1·69 (1·05-2·74) | 0·031 |
| **Work-Life balance** _[3]_ |  |  |  |  |  |  |  |
| Bottom tertile | 67 (39·6) | 95 (30·7) | 50 (17·5) | Ref. |  | Ref. |  |
| Middle tertile | 61 (36·1) | 115 (37·2) | 82 (28·8) | 1·49 (1·06-2·08) | 0·021 | 1·15 (0·80-1·66) | 0·451 |
| Top tertile | 41 (24·3) | 99 (32·1) | 153 (53·7) | 3·29 (2·34-4·63) | <0·001 | 1·88 (1·27-2·79) | 0·002 |
| **Main job task** |  |  |  |  |  |  |  |
| Physical work with hands | 34 (20·1) | 56 (18·1) | 36 (12·6) | Ref. |  |  |  |
| Mental work with figures/symbols | 50 (29·6) | 89 (28·8) | 62 (21·7) | 1·11 (0·74-1·68) | 0·609 |  |  |
| Contact/Communication with other people | 56 (33·1) | 76 (24·6) | 54 (19·0) | 0·94 (0·62-1·42) | 0·759 |  |  |
| Not working | 29 (17·2) | 88 (28·5) | 133 (46·7) | 2·79 (1·86-4·19) | <0·001 |  |  |
| **Political preference/involvement** (last elections) |  |  |  |  |  |  |  |
| Did not vote | 98 (58·0) | 146 (47·3) | 86 (30·2) | Ref. |  | Ref. |  |
| Opposition parties | 14 (8·3) | 30 (9·7) | 35 (12·3) | 2·14 (1·34-3·40) | 0·001 | 1·50 (0·91-2·47) | 0·110 |
| Governing parties | 57 (33·7) | 133 (43·0) | 164 (57·5) | 2·34 (1·76-3·12) | <0·001 | 1·41 (1·03-1·93) | 0·033 |
| **Participation at religious meetings** |  |  |  |  |  |  |  |
| At least once a month | 25 (14·8) | 40 (12·0) | 41 (14·4) | Ref. |  | Ref. |  |
| Less than once a month | 32 (18·9) | 48 (15·5) | 41 (14·4) | 0·82 (0·51-1·34) | 0·439 |  |  |
| Never, or almost never | 112 (66·3) | 221 (71·5) | 203 (71·2) | 1·04 (0·70-1·53) | 0·856 |  |  |
| **Contact with a close person (except children) I can talk to** |  |  |  |  |  |  |  |
| Less than once a week | 18 (10·7) | 37 (12·0) | 20 (7·0) | Ref. |  |  |  |
| At least once a week | 33 (19·5) | 56 (18·1) | 62 (21·8) | 1·52 (0·91-2·52) | 0·106 |  |  |
| Daily | 118 (69·8) | 216 (69·9) | 203 (71·2) | 1·38 (0·89-2·14) | 0·145 |  |  |
| **In conversations I consider myself a:** |  |  |  |  |  |  |  |
| *“No, but…” type* | 52 (30·8) | 81 (26·2) | 65 (22·8) | Ref. |  |  |  |
| *“Yes, and…” type* | 117 (69·2) | 228 (73·8) | 220 (77·2) | 1·33 (0·99-1·80) | 0·064 |  |  |
| **Optimism** _[4]_ |  |  |  |  |  |  |  |
| Bottom tertile | 61 (36·1) | 113 (36·6) | 67 (23·5) | Ref. |  |  |  |
| Middle tertile | 53 (31·4) | 78 (25·2) | 81 (28·4) | 1·32 (0·93-1·85) | 0·116 |  |  |
| Top tertile | 55 (32·5) | 118 (38·2) | 137 (48·1) | 1·82 (1·33-2·49) | <0·001 |  |  |
| **Interpersonal trust** _[5]_ |  |  |  |  |  |  |  |
| Bottom tertile | 38 (22·5) | 52 (16·8) | 49 (17·2) | Ref. |  |  |  |
| Middle tertile | 83 (49·1) | 139 (45·0) | 107 (37·5) | 0·98 (0·68-1·42) | 0·909 |  |  |
| Top tertile | 48 (28·4) | 118 (38·2) | 129 (45·3) | 1·61 (1·10-2·35) | 0·014 |  |  |
| **Empathy** _[6]_ |  |  |  |  |  |  |  |
| Bottom tertile | 57 (33·7) | 82 (26·5) | 74 (26·0) | Ref. |  |  |  |
| Middle tertile | 53 (31·4) | 114 (36·9) | 81 (28·4) | 1·07 (0·76-1·50) | 0·695 |  |  |
| Top tertile | 59 (34·9) | 113 (36·6) | 130 (45·6) | 1·47 (1·06-2·05) | 0·021 |  |  |
| **Perspective taking** _[6]_ |  |  |  |  |  |  |  |
| Bottom tertile | 65 (38·5) | 90 (29·1) | 76 (26·7) | Ref. |  | Ref. |  |
| Middle tertile | 45 (26·6) | 97 (31·4) | 61 (21·4) | 1·07 (0·75-1·51) | 0·710 | 0·99 (0·69-1·44) | 0·974 |
| Top tertile | 59 (34·9) | 122 (39·5) | 148 (51·9) | 1·75 (1·27-2·40) | 0·001 | 1·45 (1·03-2·03) | 0·033 |
| **Conscientiousness** _[7]_ |  |  |  |  |  |  |  |
| Bottom tertile | 69 (40·8) | 119 (38·5) | 74 (26·0) | Ref. |  |  |  |
| Middle tertile | 44 (26·1) | 93 (30·1) | 95 (33·3) | 1·64 (1·18-2·27) | 0·003 |  |  |
| Top tertile | 56 (33·1) | 97 (31·4) | 116 (40·7) | 1·68 (1·22-2·31) | 0·001 |  |  |
| **Extroversion** _[7]_ |  |  |  |  |  |  |  |
| Bottom tertile | 54 (31·9) | 97 (31·4) | 79 (27·7) | Ref. |  |  |  |
| Middle tertile | 62 (36·7) | 114 (36·9) | 90 (31·6) | 0·99 (0·72-1·37) | 0·954 |  |  |
| Top tertile | 53 (31·4) | 98 (31·7) | 116 (40·7) | 1·39 (1·00-1·93) | 0·051 |  |  |
| **Agreeableness** _[7]_ |  |  |  |  |  |  |  |
| Bottom tertile | 62 (36·7) | 113 (36·6) | 80 (28·1) | Ref. |  |  |  |
| Middle tertile | 68 (40·2) | 117 (37·8) | 90 (31·6) | 1·03 (0·75-1·40) | 0·876 |  |  |
| Top tertile | 39 (23·1) | 79 (25·6) | 115 (40·3) | 1·94 (1·39-2·72) | <0·001 |  |  |
| **Openness** _[7]_ |  |  |  |  |  |  |  |
| Bottom tertile | 78 (46·1) | 111 (35·9) | 90 (31·6) | Ref. |  |  |  |
| Middle tertile | 50 (29·6) | 95 (30·8) | 83 (29·1) | 1·28 (0·92-1·77) | 0·139 |  |  |
| Top tertile | 41 (24·3) | 103 (33·3) | 112 (39·3) | 1·77 (1·29-2·43) | <0·001 |  |  |
| **Neuroticism** _[7]_ |  |  |  |  |  |  |  |
| Bottom tertile | 37 (21·9) | 85 (27·5) | 106 (37·2) | Ref. |  |  |  |
| Middle tertile | 41 (24·3) | 60 (19·4) | 49 (17·2) | 0·54 (0·36-0·79) | 0·002 |  |  |
| Top tertile | 91 (53·8) | 164 (53·1) | 130 (45·6) | 0·60 (0·44-0·82) | 0·001 |  |  |
| **COVID-19 infection (positive test)** | 11 (6·5) | 23 (7·4) | 14 (4·9) | 0·79 (0·46-1·33) | 0·372 |  |  |
| **Approval of the COVID-19 measures implemented by the government** |  |  |  |  |  |  |  |
| No, they were unnecessary/ unjustified | 19 (11·2) | 12 (3·9) | 7 (2·5) | Ref. |  | Ref. |  |
| Yes, partially | 80 (47·4) | 112 (36·2) | 50 (17·5) | 1·73 (0·89-3·35) | 0·105 | 1·56 (0·76-3·21) | 0·230 |
| Yes, mainly or totally | 70 (41·4) | 185 (59·9) | 228 (80·0) | 5·45 (2·84-10·4) | <0·001 | 3·36 (1·64-6·88) | 0·001 |
| **Probability that friends/acquaintances are already vaccinated or will get vaccinated** |  |  |  |  |  |  |  |
| Almost all or all are already ··vaccinated | 10 (5·9) | 19 (6·2) | 22 (7·7) | Ref. |  | Ref. |  |
| Very likely | 31 (18·3) | 86 (27·8) | 133 (46·7) | 1·58 (0·88-2·80) | 0·120 | 1·30 (070-2·39) | 0·404 |
| Rather likely | 47 (27·8) | 95 (30·7) | 82 (28·8) | 0·80 (0·45-1·43) | 0·459 | 0·76 (0·41-1·39) | 0·369 |
| Nether likely nor unlikely | 68 (40·2) | 97 (31·4) | 42 (14·8) | 0·40 (0·22-0·72) | 0·002 | 0·51 (0·27-0·94) | 0·030 |
| Unlikely | 10 (5·9) | 8 (2·6) | 5 (1·7) | 0·31 (0·12-0·79) | 0·014 | 0·33 (0·12-0·88) | 0·027 |
| Very unlikely | 3 (1·7) | 4 (1·3) | 1 (0·3) | 0·30 (0·08-1·18) | 0·084 | 0·27 (0·06-1·22) | 0·090 |
| **BMI** [kg/m²] |  |  |  |  |  |  |  |
| Normal weight [BMI≥18·5 & <25] | 74 (49·7) | 130 (46·8) | 116 (43·9) | Ref. |  |  |  |
| Underweight [BMI<18·5] | 7 (4·7) | 9 (3·2) | 4 (1·5) | 0·51 (0·22-1·16) | 0·109 |  |  |
| Overweight [BMI≥25 & <30] | 43 (28·8) | 89 (32·0) | 92 (34·9) | 1·24 (0·90-1·71) | 0·184 |  |  |
| Obesity [BMI≥30] | 25 (16·8) | 50 (18·0) | 52 (19·7) | 1·22 (0·83-1·80) | 0·302 |  |  |
| **Frequency of physical activity done for at least 10 minutes which raises the heartbeat or the respiratory rate** |  |  |  |  |  |  |  |
| Less than once a week | 26 (15·4) | 42 (13·6) | 43 (15·1) | Ref. |  |  |  |
| 1-2 days a week | 49 (29·0) | 93 (30·1) | 72 (25·3) | 0·89 (0·58-1·35) | 0·570 |  |  |
| 3-4 days a week | 54 (32·0) | 86 (27·8) | 73 (25·6) | 0·85 (0·55-1·31) | 0·457 |  |  |
| 5-7 days a week | 40 (23·6) | 88 (28·5) | 97 (34·0) | 1·27 (0·83-1·95) | 0·268 |  |  |
| **Smoking status** |  |  |  |  |  |  |  |
| Never | 80 (47·3) | 136 (44·0) | 113 (39·6) | Ref. |  |  |  |
| Former | 38 (22·5) | 82 (26·5) | 84 (29·5) | 1·36 (0·98-1·88) | 0·065 |  |  |
| Current | 51 (30·2) | 91 (29·5) | 88 (30·9) | 1·16 (0·85-1·59) | 0·344 |  |  |
| **Chronic disease** _[8]_ | 47 (27·8) | 113 (36·6) | 148 (51·9) | 2·11 (1·60-2·78) | <0·001 | 1·59 (1·16-2·16) | 0·003 |
| [1] mutually adjusted for all variables for which adjusted odds ratios with 95% confidence intervals and adjusted p-values are reported [2] household income tertiles were approximated because the distributions did not permit precise tertile cut-points [3] TKS-WLB^29^ [4] LOT-R^30^ [5] KUSIV3^31^ [6] questionnaire for empathy and perspective taking, German version^32^ [7] BFI-S^33^ [8] asthma, COPD, chronical bronchitis, emphysema, heart attack, angina pectoris or coronary heart disease, cancer, hypertension, stroke or diabetes | | | | | | | |
